# Biofilm mediated synergistic degradation of hexadecane by a naturally formed community comprising *Aspergillus flavus complex* and *Bacillus cereus* group

**DOI:** 10.1186/s12866-019-1460-4

**Published:** 2019-04-29

**Authors:** Madushika Perera, Dilrukshi Wijayarathna, Sulochana Wijesundera, Manoj Chinthaka, Gamini Seneviratne, Sharmila Jayasena

**Affiliations:** 1 Department of Biochemistry and Molecular Biology, Faculty of Medicine, University of Colombo, Colombo 08, Sri Lanka; 2 Department of Chemistry, Faculty of Science, University of Colombo, Colombo, Sri Lanka; 3 Department of Chemistry, Faculty of Applied Sciences, University of Sri Jayewardenepura, Nugegoda, Sri Lanka; 4 National Institute of Fundamental Studies, Hantana Road, Kandy, Sri Lanka

**Keywords:** *Aspergillus*, *Bacillus*, Biofilm, Synergism, Hydrocarbon, Hexadecane, Biodegradation, Microbial community

## Abstract

**Background:**

The hydrophobic nature of hydrocarbons make them less bioavailable to microbes, generally leading to low efficiency in biodegradation. Current bioremediation strategies for hydrocarbon contamination, uses induced mixed microbial cultures. This in-vitro study demonstrates the utilization of naturally occurring communities in suitable habitats for achieving highly efficient, synergistic degradation of hydrocarbons in a simple community structure without additives.

**Methods:**

Enrichment media supplemented with 1% (7652.53 mg/L) hexadecane (HXD) as the sole carbon source were inoculated with samples of soil with waste polythene, collected from a municipal landfill in order to isolate microbial communities. Gas Chromatography-Mass Spectrometry (GC-MS) analysis was performed on HXD grown co-cultures and individual counterparts after 14 days incubation and percentage degradation was calculated. Microbes were identified using 16S rRNA gene and Internal Transcribed Spacer region sequencing. Biofilm formation was confirmed through scanning electron microscopy, in the most efficient community.

**Results:**

Three mixed communities (C1, C2 and C3) that demonstrated efficient visual disintegration of the HXD layer in the static liquid cultures were isolated. The C1 community showed the highest activity, degrading > 99% HXD within 14 days. C1 comprised of a single fungus and a bacterium and they were identified as a *Bacillus* sp. MM1 and an *Apsergillus* sp. MM1. The co-culture and individual counterparts of the C1 community were assayed for HXD degradation by GC-MS. Degradation by the fungal and bacterial monocultures were 52.92 ± 8.81% and 9.62 ± 0.71% respectively, compared to 99.42 ± 0.38% by the co-culture in 14 days. This proved the synergistic behavior of the community. Further, this community demonstrated the formation of a biofilm in oil-water interface in the liquid medium. This was evidenced from scanning electron microscopy (SEM) showing the *Bacillus* cells attached on to *Aspergillus* mycelia.

**Conclusions:**

This study demonstrates the utilization of naturally formed fungal-bacterial communities for enhanced biodegradation of hydrocarbons such as hexadecane and reports for the first time, synergistic degradation of hexadecane through biofilm formation, by a community comprising of *Bacillus cereus* group and *Aspergillus flavus* complex.

**Electronic supplementary material:**

The online version of this article (10.1186/s12866-019-1460-4) contains supplementary material, which is available to authorized users.

## Background

Microbial community mediated degradation of environmental pollutants is a promising application over exploiting the genetic potential of single microbes to clean up contaminants. Although this single microbe technology has been exploited in the past for its potential for bioremediation, there are limitations pertaining to the bioavailability and recalcitrant nature of contaminants.

Hydrocarbon contamination in aquatic as well as in terrestrial habitats are one of the major concerns worldwide. Aliphatic and aromatic fractions of hydrocarbons are both found in the contaminated sites. Single organisms of various microbial genera have been reported [1] to degrade these hydrocarbons. However, the hydrophobic nature of hydrocarbons makes it less bioavailable to microbes, hence they persist in the environment for extended periods of time. These complex organic contaminants are therefore not easily mineralized as no organism can alone remove them.

Single/pure cultures of microbes in planktonic mode or as biofilms have been of great interest over the last few decades, with regard to biodegradation of contaminants [2, 3]. In laboratory conditions, pure cultures obtained from environmental samples are tested for contaminant degradation considering only abiotic factors, without due consideration of the nature of its natural habitat; whether it is present as a single organism, as planktonic cells, or as biofilms or it is adapted to a community mode of mixed organisms. Therefore, exploitation of an organism’s full potential in removal of contaminants by manipulating its environment requires that its community behavior is taken in to consideration, even in the laboratory applications.

In nature, microbes interact with all biotic and abiotic factors and maintain its persistence through balancing the synergistic and antagonistic effects. Synergism allows microbes to thrive in various habitats through adaptation. A community of microbes consisting of multiple species contains an increased repertoire of genes and metabolic capabilities compared to monocultures. Thus, if one organism alone could not achieve the complete utilization of a particular substrate, it can harness the potential of other members of the community in order to maximize the output of the process. This is described as the division of labor across organisms in natural communities [4].

Fungal-bacterial interactions address both bioavailability and bioaccessability which otherwise hinder the initial oxidation of contaminants in the process of mineralization. Bacterial biofilms on fungal surfaces is a special form of biofilms where bacterial cells attach to fungal hyphae which is a biotic surface, rather than attaching on to abiotic surfaces [5]. Fungal hyphae are not merely a surface to support the attachment but play multiple roles. They provide a source of nutrients for bacteria, enable bacteria to “travel” with hyphae in the available medium in search of nutrients [6] providing bioaccessability to bacteria and increase acquisition of nutrients by competition etc. Soil associated penanthrene has been shown to be degraded by motile *Pseudomonas putida* PpG7 only in the presence of *Pythium ultimum* fungal mycelia [7].

Various chemical stimuli released by fungi attract bacteria by chemotactic response. *Bacillus subtilis* has been reported to migrate through soil toward fungal spores as a chemotactic response, to exudates released from them [8]. Expression of specific genes upon contact with fungal cells [9, 10] is another advantage of fungal - bacterial biofilms. Several *P. putida* genes (an uncharacterized transcription factor, an ABC transporter, and a porin) have been reported to be induced during growth on the surface of the fungus *Phytophthora parasitica* [11] *P. fluorescens* has been shown to induce its trehalose utilization genes when exposed to fungal culture supernatant [12]. Trehalose is a sugar commonly found in fungi which acts as a chemo attractant [13]. In favor of biofilm formation on fungal hyphae, bacteria provide extracellular polymeric substances (EPS), facilitating the attachment of cells [14]. Bacterial motility greatly influences them to reach surfaces suitable for formation of biofilm and flagella help to adhere on to the surface [15, 16]. *B. cereus* and *B. thuringiensis* adhere on to biotic surfaces through their flagella [17].

The aim of this study was to investigate the interaction between *Bacillus* sp. and *Aspergillus* sp. growing in HXD as the sole carbon source for possible synergistic activity and to examine the mode of interaction of the two organisms in the co-culture.

## Methods

### Collection of samples

Samples of waste polythene (with attached soil) were collected from a municipal landfill (Meethotamulla, Kolonnawa) in Colombo district, Sri Lanka. Samples were collected (in to sterile containers) at a depth of 1–6 cm from soil surface, in order to acquire an aerobic microbial population [18]. The temperature of the collection spot was 42 °C. Sample pH was 9.5. Collected samples were stored at 4 °C.

### Culture conditions

Nutrient broth (NB) or nutrient agar (NA) and Sabouraud dextrose broth (SDB) or Sabouraud dextrose agar (SDA) media (Hardy Diagnostics) were used to isolate and maintain bacterial and fungal cultures respectively. Bushnell and Haas (BH) medium [19], MgSO_4_ 0.2 g/L, CaCl_2_ 0.02 g/L, KH_2_PO_4_ 1.0 g/L, K_2_HPO_4_ 1.0 g/L, (NH_4_)_2_SO_4_ 1.0 g/L, FeCl_3_ 0.05 g/L, 6.8 pH, was used in assays which were conducted to measure the ability of microbes to utilize a given source of carbon and energy. Filter sterilized (0.22 μm pore size) n-hexadecane (C_16_H_34_) (HXD) (Sigma-Aldrich), crude oil (obtained from Ceylon Petroleum Corporation (CEYPETCO) refinery), diesel and petrol (obtained from a CEYPETCO filling station - Colombo, Sri Lanka) were individually used as the sole source of carbon and energy in BH medium at a concentration of 1% (v/v). The incubation temperature was set at 40 °C. Experiments were carried out under aerobic conditions (without agitation to enhance the microbial growth in the form of a biofilm) unless otherwise specified. All assays were conducted in triplicate and with a negative control (under same conditions, without microbial inoculum).

### Culture enrichment and isolation of microbes

NB (100 mL) was inoculated with 1 g of each of the collected samples and incubated for 16 h on a rotary shaker (180 rpm). Culture enrichment was carried out in BH medium (100 mL) supplemented with 1% HXD. Enrichment medium was inoculated with 1% of the NB culture and incubated until microbial growth was observed (approximately three weeks). The turbid broth was serially diluted and spread on NA and incubated for 16 h. Well separated single colonies were streaked on NA to obtain pure bacterial cultures.

### Screening and selection of isolates for further studies

The bacterial isolates obtained above were grown on BH medium supplemented with 1% HXD individually and re-screened for HXD degradation, based on the visual disintegration of the HXD layer with oil droplets on the medium.

### Isolation of community counterparts

Naturally formed mixed communities (C1, C2, C3) that appeared during screening were grown on both NA to (promote bacterial growth) and SDA (to promote fungal growth) in order to identify the participating bacteria and fungi in each community. Bacteria were purified as mentioned above and fungi were purified using single spore isolation technique [20]. Finally they were preserved in glycerol and stored in – 80 °C [21].

### Identification of microbes in the community C1

Identification of bacteria was performed by microscopic, biochemical and molecular techniques. Gram’s staining and biochemical tests were performed according to the Bergey’s Manual of Systematic Bacteriology [22]. For molecular identification, genomic DNA was extracted using Wizard Genomic DNA Purification Kit (Promega) according to the manufacturer’s instructions. The 16 s rRNA gene was amplified by polymerase chain reaction (PCR) with universal primers; Primer forward (27F); 5′ AGAGTTTGATCMTGGCTCAG 3′ and primer reverse; (1492R) 5′ GTA TTA CCG CGG CTGCTGG 3′ [23]. The following thermal profile was used; initial denaturation at 95 °C for 5 min, 30 cycles of denaturation at 95 °C for 30 s, annealing at 55 °C for 30 s and extension at 72 °C for 90 s and a final extension at 72 °C for 10 min. The amplified gene was sequenced (Macrogen Co. South Korea (ABI PRISM 3730XL Analyzer-96 capillary type). The obtained sequence was analyzed by Basic Local Alignment Search Tool (BLAST), http://www.ncbi.nlm.nih.gov/blast, National Center for Biotechnology Information (NCBI), to identify the organism.

Fungal identification was carried out by macroscopic observation of colony morphology and microscopic morphology with the aid of slide culture technique with lacto phenol cotton blue (LPCB) staining. Molecular identification of the fungus was based on the analysis of the Internal Transcribed Spacer (ITS) region sequence. Fungal genomic DNA was isolated [24]. The ITS region was PCR - amplified with universal primers, ITS1 (5′ TCCGTAGGTGAACCTGCGG 3′) and ITS4 (5′ TCCTCCGCTTATTGATATGC 3′) [25] using the following thermal profile; initial denaturation at 95 °C for 10 min, 30 cycles of denaturation at 95 °C for 1 min, annealing at 55 °C for 1 min and extension at 72 °C for 90 s and a final extension at 72 °C for 10 min. Sequence analysis was as described above.

### Analysis of HXD biodegradation of co-cultures and individual counterparts by GC-MS

Co-cultures and individual counterparts were assayed for HXD biodegradation. Erlenmeyer flasks (100 mL) containing 20 mL BH medium with 1% HXD (7652.53 mg/L) was inoculated (5*10^4^ bacterial cells/mL, 5*10^3^ fungal spores/mL) and incubated continuously for 14 days. Control flasks were incubated under identical conditions but devoid of the microbial inoculum (negative control).

At the end of 14 days of incubation, residual HXD in the growth medium was extracted four times with hexane (5 mL) and the combined solvent extract was dried using anhydrous sodium sulfate (Na_2_SO_4_). HXD in hexane was then quantified chromatographically via Gas chromatography-Mass spectrometry (GC–MS) using Agilent 7890a GC system equipped with 5975c MS system, split injector, and a capillary column (Agilent 19,091 s-33HP-5MS 5% Phenyl Methyl Silox; 30 m × 250 μm × 0.25 μm). The optimized temperature program was as follows; oven temperature 50 °C for 5 min, then increased to 250 °C at a rate of 20 °C/min and kept at 250 °C for 1 min where the total run time was 16 min. The injector temperature was maintained at 270 °C. The carrier gas used in the column was helium at a flow rate of 1.5 mL/min [26]. Chromatographic peak for HXD was obtained at the retention time of 12.66 min.

The chromatogram obtained by GC-MS analysis showed the relative abundance of HXD in the hexane extract. The biotic degradation of the HXD was obtained from the difference in the area of HXD peak of the sample and the control, and percentage degradation was calculated.

### Scanning Electron microscopic (SEM) observation of C1

The scrap of the mature biofilm (after 14 days) was transferred onto an EM stub and allowed to air dry (approx. 30 min). The sample was examined under a scanning electron microscope (TESCAN VEGA3).

### Degradation of other hydrocarbons

Erlenmeyer flasks (100 mL) containing 20 mL BH medium supplemented with 1% of filter-sterilized crude oil, diesel or petrol as the sole carbon source respectively, were inoculated with C1 community and incubated continuously for 14 days (40 °C without agitation). Crude oil degradation was observed visually. Degradation of petrol and diesel was detected using Dichlorophenolindophenol (DCPIP). Filter sterilized DCPIP solution was added to the medium to detect the utilization of the carbon source by the color change of the medium, from bright blue to colorless [27].

### Statistical analysis

One-way ANOVA and multiple comparisons of means by using Tukey’s HSD test in the SPSS v.18.0 (SPSS Inc.; IBM Company; Chicago, IL, USA) statistical package were applied to determine the statistical significance (at *p* < 0.05) of the percentage degradation of HXD by the three communities. All data are presented as means ± standard deviation of triplicates (*n* = 3).

## Results

### Culture enrichment and isolation of microbes

A mixed community of bacteria was obtained from samples (waste polythene attached with soil) collected from the municipal landfill. Ten well separated bacterial colonies were isolated and preserved.

### Screening and selection of isolates

Ten bacterial isolates were grown in 1% HXD in planktonic mode, where the turbidity was observed throughout the culture medium. Among the ten bacterial isolates, three were subsequently observed to form mixed communities with fungi, where mycelial growth was observed on the liquid medium, shifting the community structure towards the biofilm mode, reducing the turbidity of planktonic bacterial cells in the medium. Further, this development was concomitant with the disintegration of the HXD layer resulting in formation of noticeable oil droplets, compared to the rest of the isolates. Therefore, these three mixed communities (C1, C2, C3) were selected for further study.

### Isolation of community counterparts

The three bacteria associated with each community had similar colony characteristics. Macroscopic identification of the fungal component in each community revealed that while C1 and C2 bacterial isolates were associated with a single fungus, C3 comprised of three fungi along with its bacterium.

### Identification of microbes in the selected community

Grams staining and biochemical characterization identified the bacterium in C1 as a Gram’s positive, active motile, endospore forming, rod shaped facultative anaerobe (Table 1).

**Table 1:** Biochemical characterization of the bacterium in C1, C2, and C3

	Bacterial isolates in the isolated communities		
Characteristics	C1- bacterium	C2- bacterium	C3- bacterium
Gram’s reaction	+	+	+
Motility	Active motile	Non-motile	motile
Endospores	+	+	+
position	Sub terminal	Sub terminal	Sub terminal
shape	Oval	Oval	Oval
swollen/no swelling	Swollen	No swelling	No swelling
Cell shape	Rods	Rods	Rods
Oxygen relationship	Facultative anaerobe	Facultative anaerobe	Facultative anaerobe
Catalase (aerobe)	+	+	+
Oxidase (aerobe)	+	+	+
Nitrate reduction (anaerobe)	+	+	+
Voges-proskuer test	–	–	–
Glucose fermentation			
gas production	–	–	–
acid production	+	+	+
Fermentation of sugars			
L- arabinose	–	–	+
D-xylose	–	–	+
D-mannitol	–	–	–
D-mannose	–	+	–
Casein hydrolysis	+	+	+
Gelatin hydrolysis	+	+	+
Starch hydrolysis	–	–	+
Citrate utilization	+	+	+
Tyrosine degradation	–	–	–
Lecithinase activity	–	–	–
Indole test	–	–	–
Growth at pH			
6.8	+	+	+
5.7	–	–	–
Growth in NaCl			
7%	+	+	+
10%	+	+	+
Growth at temperatures			
30 °C	+	+	+

BLAST analysis of the nucleotide sequence of 16 s rRNA gene of the bacterium showed the maximum identity with *B. cereus* group bacteria, *B. cereus*, *B. thuringiensis* and *B. anthracis*, revealing that it is difficult to differentiate between them [28–31]. *B. anthracis* is non-motile. Since, bacterium in C1 community of our study is an active motile strain (Table 1) it was concluded to be a *B. cereus*/*B. thuringiensis* (*Bacillus* sp. MM1; GenBank accession no. MH503924).

The fungus in C1 grown on SDA plates for 3 to 5 days exhibited morphological features as follows; white velvety colonies with floccose texture changed during sporulation in to a greenish-gray with white edges on the anverse side and light brown on the reverse side. Slide cultures of the fungal isolate were prepared on SDA medium by growing for 3 to 5 days, for microscopic observation. BLAST analysis of the nucleotide sequence of the 416 bp fragment of ITS region revealed the fungus belongs to the genus *Aspergillus* sp. (*Aspergillus* sp. MM1; GenBank accession no. MH503926). Further, 100% homology was found with *A. flavus* complex fungi, *A. flavus* and *A. oryzae* sequences, reported in GenBank (NCBI). Species differentiation of the fungus of the C1 community between *A. flavus* and *A. oryzae* has also been shown to be controversial [30, 31].

### HXD biodegradation of co-cultures and individual counterparts by GC-MS

**Fig. 1:**
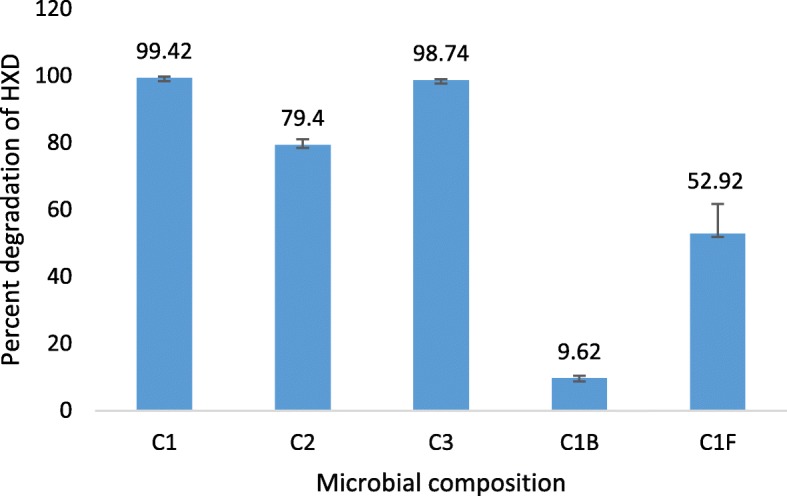
Percentage degradation of HXD after 14 days by the three microbial communities (C1, C2, C3) and counterparts of C1 (C1B; Bacterium of C1, C1F; Fungus of C1). Error bars represent standard deviations (*n* = 3), Significance of mean differences was set at *P* ≥ 0.05

Utilization of HXD as sole carbon source by C1, C2 and C3 communities were compared by GC-MS analysis of residual HXD. HXD biodegradation by the three co-cultures C1, C2 and C3 after 14 days incubation were 99.42 ± 0.38%, 79.4 ± 1.65% and 98.74 ± 0.23% respectively (Fig. 1) and difference between three communities were statistically significant (one-way ANOVA; *P* < 0.001). The HXD degradation capability of the C1 and C3 communities were both significantly higher than C2 (*P* = 0.021 and *P* = 0.025 respectively). C1 and C3 communities revealed the highest degradation activity, with no significant difference between them (*P* < 0.05). However, C1 comprised of a single bacterium and a single fungus while C3 showed the involvement of at least three species of fungi interacting with a single bacterium, forming the community. Hence, C1 was selected for investigation, considering its efficiency and simplicity of the microbial composition.

Analysis of the activity of each of the interacting organisms in C1 under the same conditions showed that HXD biodegradation by the fungal and bacterial monocultures were 52.92 ± 8.81% and 9.62 ± 0.71% respectively, while the degradation by the co-culture was 99.42 ± 0.38%. Thus, synergistic association between the fungus and the bacterium in the selected co-culture was evident (Fig. 1).

### Biofilm formation by the C1 community

**Fig. 2:**
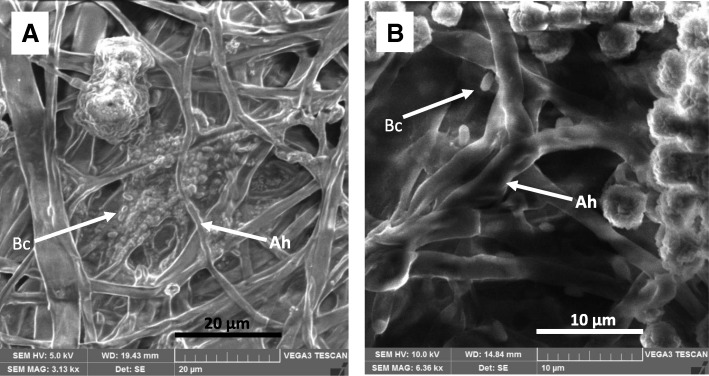
SEM micrographs of mature *Aspergillus* - *Bacillus* biofilm (14 days after co-inoculation) formed in HXD containing medium. **a** Magnification 3130X **b** magnification 6360X; the arrows point either *Aspergillus* hyphae (Ah) or *Bacillus* cells (Bc)

Light microscopic imaging of the mature community (after 14 days) grown on HXD showed visible colonization of the Bacillus cells on the *Aspergillus* mycelia. Scanning electron micrographs (SEM) further confirmed the formation of a biofilm by the *Aspergillus* - *Bacillus* community. It clearly shows the matrices of extracellular polymeric substances (EPS) which embedded the bacterial cells as well as the fungal mycelia in the mature biofilm (Fig. 2).

### Degradation of other hydrocarbons by the C1 community

**Fig. 3:**
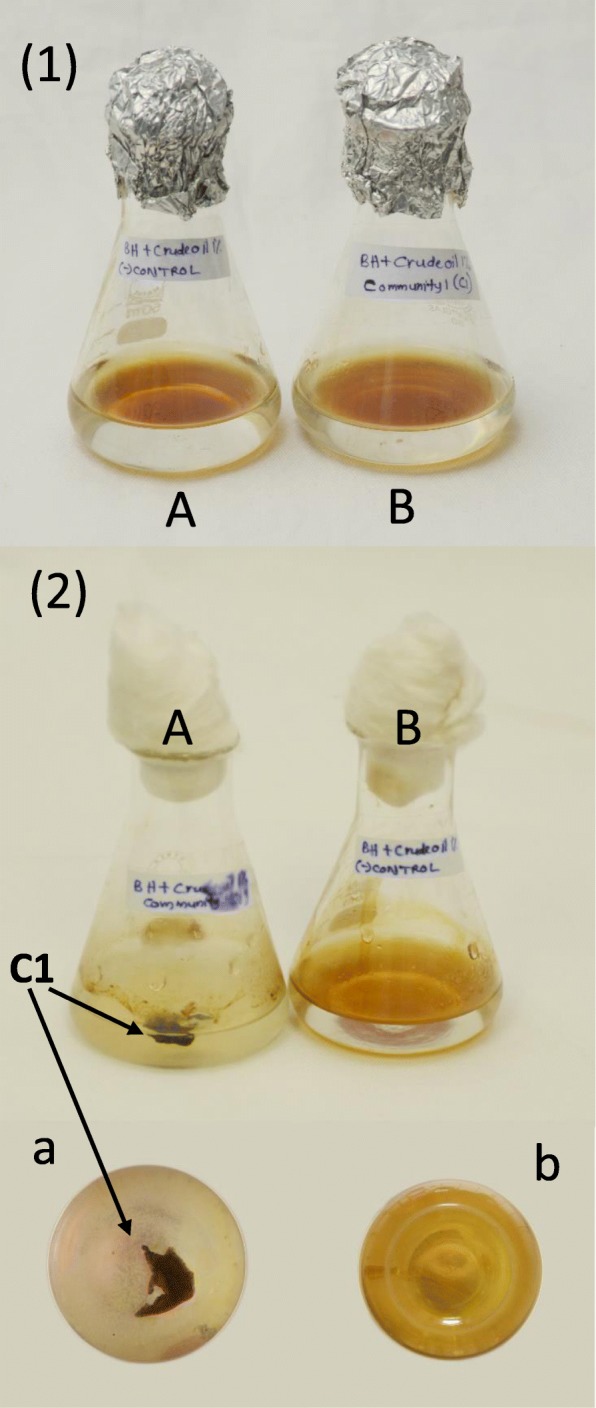
Crude oil degradation by *Aspergillus* - *Bacillus* (C1) co-culture**.** (1) 1% crude oil in BH medium before incubation (A) negative control, (B) inoculated with C1 co-culture (2) 1% crude oil in BH medium after 14 days incubation (A) inoculated with C1 co-culture, (B) negative control (a), (b) A and B flasks after 14 days as viewed from bottom

Crude oil (1%) as the sole carbon source in BH medium was visually observed to be utilized by C1 which showed the disintegration of the continuous oil layer (brown in color) in to oil droplets (Fig. 3). The DCPIP assay results confirmed the ability of C1 to utilize diesel as the sole carbon source (1%) in the BH medium. But 1% petrol as sole carbon source in BH medium did not give a significant color change to DCPIP in the medium after 14 days, indicating that C1 is unable to utilize petrol.

## Discussion

Mixed microbial communities such as those consisting of fungi and bacteria, can potentially provide an advantage for survival in an environment where both bioavailability and bio-accessibility of carbon sources are low. The symbiosis enables microbes to adapt to a new environment, survive in nutrient limiting conditions and further acquire metabolic alterations in order to thrive [32–35]. It also provides a potential for synergism.

In the present study, three natural fungal-bacterial communities which possess the ability to degrade hydrocarbons (under aerobic conditions) were isolated from Meethotamulla, a landfill in Colombo municipal area, Sri Lanka. Biodegradation of HXD in 14 days increased from 52.92 ± 8.81% and 9.62 ± 0.71% in the fungal and bacterial monocultures respectively, to 99.42 ± 0.38% when in co-culture. This clearly demonstrated synergistic degradation of HXD as the sole carbon source by this naturally occurring C1 community in liquid medium.

C1 contains a bacterium from *B. cereus* group and a fungus from *A. flavus* complex. Several mixed microbial communities have been recorded in hydrocarbon degradation, such as *Mycobacterium hyalinum* and *Cladosporium* degrading diesel oil [34] and biodegradation of oil contaminated soil by a yeast - bacterial co-culture [35]. Degradation of 2-naphthol has been achieved by coupling *A. niger* with *B. subtilis* [36]*.* However, *B. cereus* group bacteria and *A. flavus* complex fungi have not been previously shown to act in a synergistic manner. Instead, *B. cereus* has been recorded for its use as a biocontrol agent of *A. flavus* due to their antagonistic behaviour [37].

Filamentous fungi are capable of producing hydrophobic proteins, enabling them to attach on to hydrophobic surfaces [38]. The fungal counterpart in the C1 community was observed to form its mycelial mat at the hydrophobic - hydrophilic interface in the HXD - BH medium. This allows the bacteria to overcome the nutrient limitation in submerged growth by attaching on to the fungal mycelium and gaining access to the hydrocarbon layer. The bacterium of C1 is an active motile strain (Table 1) and would thus be capable of travelling through the medium up to the fungal mat. Bacterial flagella motility is recorded to influence the movement towards a target surface and attachment to it [15, 16] .

In the present study, proved synergistic association between the fungus and the bacterium of the C1 co-culture, leads to a hypothesis of a possible biofilm mode for the community structure. Previous studies have provided evidence for successful association of microbial communities consisting of more than one species, overcoming the need for an abiotic surface for the biofilm formation [2, 9, 39, 40].

Scanning electron microscopic images of the C1 community (Fig. 2) confirmed the presence of the biofilm formation of the community interaction where *Aspergillus* hyphae is colonized by *Bacillus* cells and both embedded in an EPS matrix. Further, the bacterium of C1 being a facultative anaerobe as shown by biochemical analysis (Table 1), explains its ability to go in to a biofilm mode from its planktonic mode.

Genes coding for enzymes involved in aerobic respiration in *B. subtilis* are down-regulated upon its attachment to *A. niger* hyphae, allowing the bacterial metabolism to be shifted from aerobic to anaerobic [10]. This supports the idea of planktonic bacterial cells going in to the biofilm mode, attaching to hyphae and further being embedded in the EPS matrices, thus being exposed to an anaerobic environment.

Further, the C1 community in biofilm mode demonstrated the ability to utilize crude oil and diesel (which contains cetane/hexadecane and iso-cetane). Crude oil consists of a variety of chemically distinct hydrocarbons, including paraffins (alkanes), naphthenes (cycloalkanes), and aromatics (benzene, toluene, ethyl benzene, xylene etc.) which require specific mechanisms for activation and biodegradation [41]. Accordingly our *Aspergillus - Bacillus* co-culture might be utilizing higher carbon number alkanes and also cycloalkanes and aromatic fraction of the crude oil, which needs further investigation.

Metabolic co-dependence in biofilm cells leads to halt the biofilm growth periodically and this is known as collective oscillation in biofilm growth. This benefits the community in nutrient limiting conditions [42]. The bacterium in the C1 community was shown to form endospores (Table 1). This would enable it to survive in a dormant state under nutrient limiting conditions. Since fungal spore produced for dissemination are also adapted for survival, both organisms are able to survive under prolonged stress conditions, allowing this combination to be used as a successful environmental remediation agent.

## Conclusion

In conclusion, it is evident that there are naturally formed microbial communities which can efficiently degrade hydrocarbons under aerobic conditions. Their degradation capacity of crude oil and diesel is higher than that of mono cultures of their counterparts, demonstrating the synergistic behavior in biofilm mode. The study suggests that the density of such communities in the nature is too low to have a significant effect on the hydrocarbon removal from contaminated sites. Thus, isolation, multiplication and utilization of the naturally occurring communities may provide a more superior option than induced communities for bioremediation purposes.

## Additional files

Additional file 1: Chromatograms_GCMS 1. Chromatograms for residual HXD analyzed by GC-MS after 14 day incubation of cultures of the three communities (C1,C2 & C3) and counterparts of community C1. (PDF 828 kb)

Additional file 2: Chromatograms_GCMS 2. Balance chromatograms for residual HXD analyzed by GC-MS after 14 day incubation of cultures of the three communities (C1,C2 & C3) and counterparts of community C1. (PDF 132 kb)

Additional file 3: Key to chromatograms in Additional files 1 and 2. (DOCX 12 kb)

## Abbreviations

BH: Bushnell and Haas
BLAST: Basic Local Alignment Search Tool
CEYPETCO: Ceylon Petroleum Corporation
DCPIP: Dichlorophenolindophenol
EPS: Extracellular polymeric substances
GC-MS: Gas Chromatography-Mass Spectrometry
HXD: Hexadecane
ITS: Internal Transcribed Spacer
LPCB: Lacto phenol cotton blue
NA: Nutrient agar
NB: Nutrient broth
NCBI: National Center for Biotechnology Information
PCR: Polymerase chain reaction
SDA: Sabouraud dextrose agar
SDB: Sabouraud dextrose broth
SEM: Scanning Electron Microscopy
