# Supplementary material for: Biofilm mediated synergistic degradation of hexadecane by a naturally formed community comprising Aspergillus flavus complex and Bacillus cereus group
Source: BMC Microbiol. 2019 Apr 29;19:84. doi: 10.1186/s12866-019-1460-4 (PMC6489202; doi:10.1186/s12866-019-1460-4)
Supplement: Supplementary file 1 — Chromatograms_GCMS 1. Chromatograms for residual HXD analyzed by GC-MS after 14 day incubation of cultures of the three communities (C1,C2 & C3) and counterparts of community C1. (PDF 828 kb) [file 12866_2019_1460_MOESM1_ESM.pdf]

File :C:\msdchem\1\data\Madushika\2016.05.05_MADUSHIKA_MM1R1W1.D
Operator :
Acquired : 5 May 2016 13:11 using AcqMethod Madushika.M
Instrument : UOSJP GCMSD
Sample Name:
Misc Info : ERR
Vial Number: 1

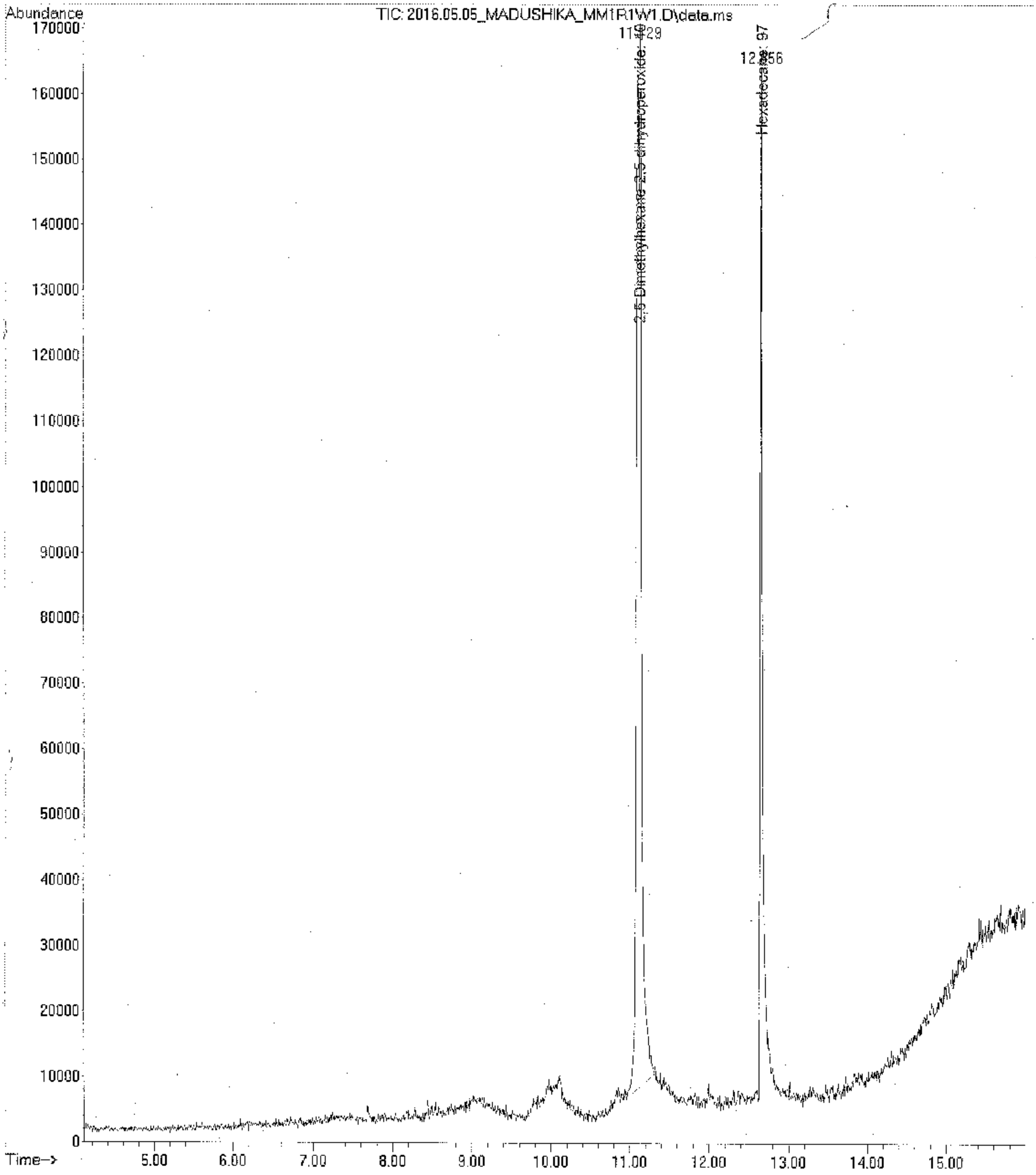

Area Percent Report

Data Path : C:\msdchem\1\data\Madushika\
Data File : 2016.05.05_MADUSHIKA_MM1R1W1.D
Acq On : 5 May 2016 13:11
Operator :
Sample :
Misc :
ALS Vial : 1 Sample Multiplier: 1

Integration Parameters: autoint1.e
Integrator: ChemStation

Method : C:\msdchem\1\methods\Calibtration plot_Ace.M
Title : autoint1.e

Signal : TIC: 2016.05.05_MADUSHIKA_MM1R1W1.D\data.ms

peak #	R.T. min	first scan	max scan	last scan	PK TY	peak height	corr. area	corr. % max.	% of total
1	11.129	1165	1193	1224	BB 4	155787	7255671	100.00%	67.728%
2	12.656	1444	1452	1479	BB	144287	3457335	47.65%	32.272%

Sum of corrected areas: 10713005

Calibtration plot_Ace.M Thu May 05 14:06:26 2016

```
File        :C:\msdchem\1\data\Madushika\2016.05.05_MADUSHIKA_MM1R2W1.D
Operator    :
Acquired    :  5 May 2016  12:31      using AcqMethod Madushika.M
Instrument  :   UOSJP GCMSD
Sample Name:
Misc Info   :                                              ERR
Vial Number: 1
```

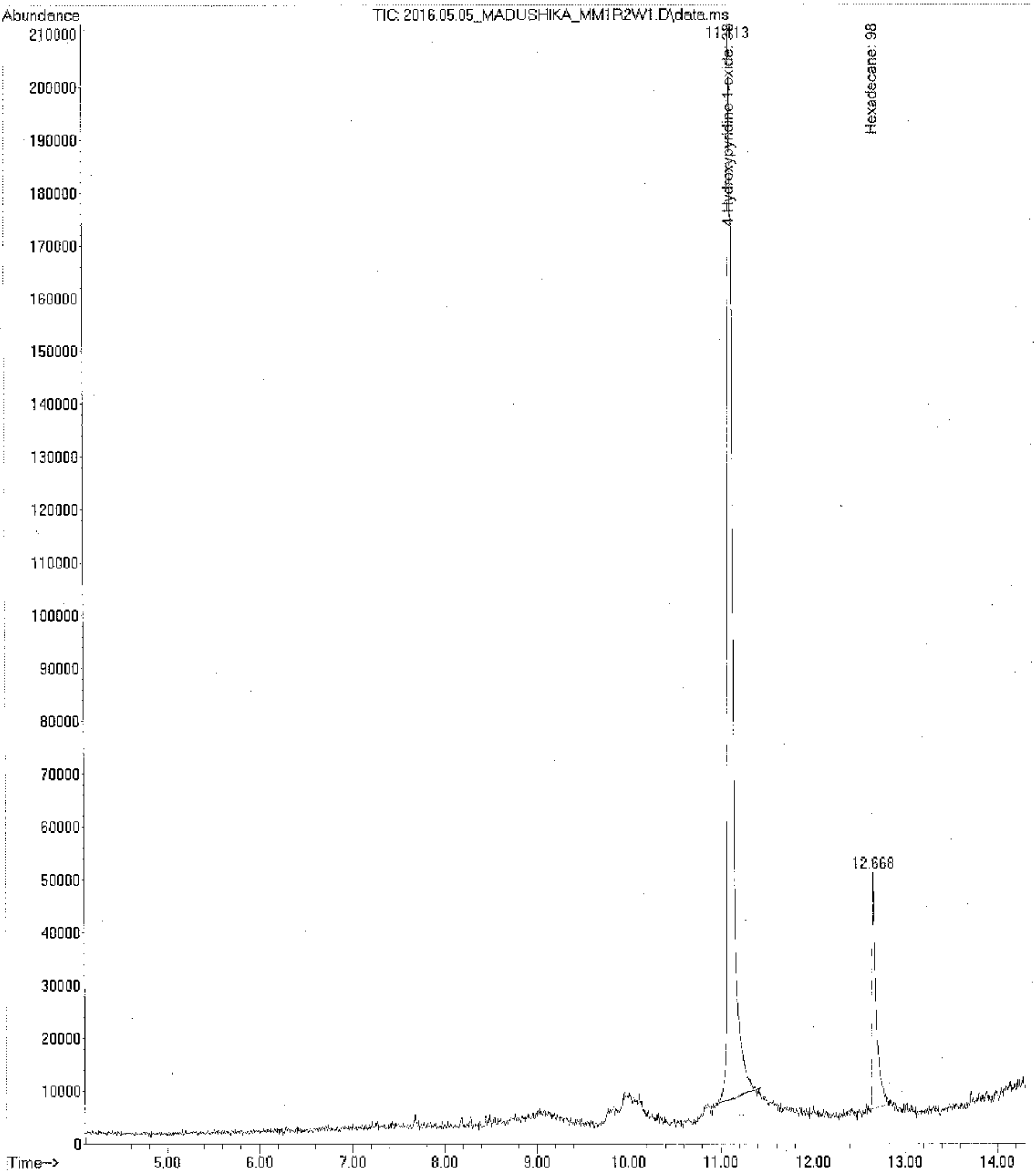

Area Percent Report

Data Path : C:\msdchem\1\data\Madushika\
Data File : 2016.05.05_MADUSHIKA_MM1R2W1.D
Acq On : 5 May 2016 12:31
Operator :
Sample :
Misc :
ALS Vial : 1 Sample Multiplier: 1

Integration Parameters: autoint1.e
Integrator: ChemStation

Method : C:\msdchem\1\methods\Calibtration plot_Ace.M
Title : autoint1.e

Signal : TIC: 2016.05.05_MADUSHIKA_MM1R2W1.D\data.ms

peak #	R.T. min	first scan	max scan	last scan	PK TY	peak height	corr. area	corr. % max.	% of total
1	11.113	1155	1190	1233	BB	195731	7978802	100.00%	86.374%
2	12.668	1430	1454	1479	BB	42734	1258721	15.78%	13.626%

Sum of corrected areas: 9237523

Calibtration plot_Ace.M Thu May 05 14:07:11 2016

File :C:\msdchem\1\data\Madushika\2016.05.05_MADUSHIKA_MM1R3W1.D
Operator :
Acquired : 5 May 2016 13:32 using AcqMethod Madushika.M
Instrument : UOSJP GCMSD
Sample Name:
Misc Info : ERR
Vial Number: 1

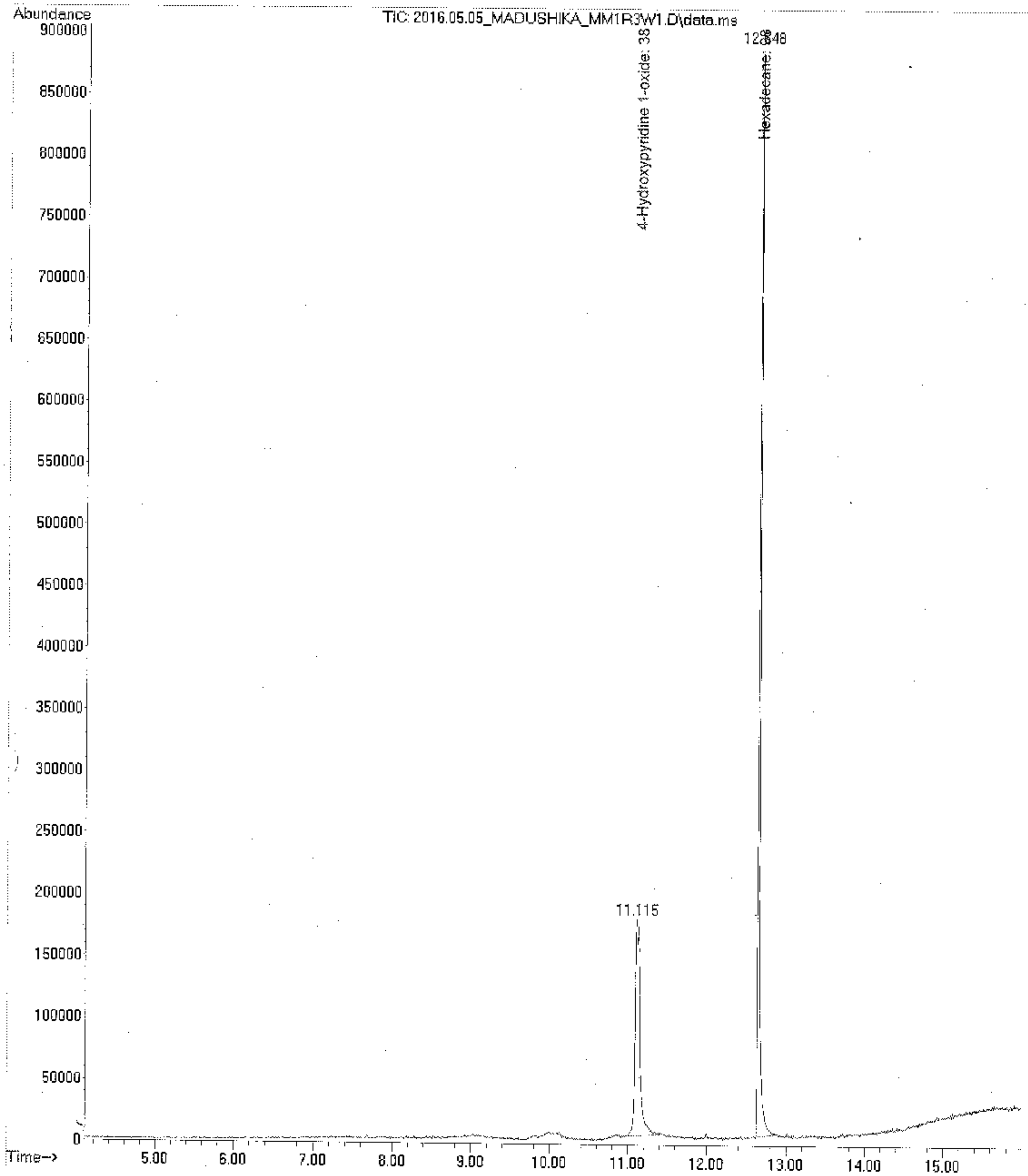

Area Percent Report

Data Path : C:\msdchem\1\data\Madushika\
Data File : 2016.05.05_MADUSHIKA_MM1R3W1.D
Acq On : 5 May 2016 13:32
Operator :
Sample :
Misc :
ALS Vial : 1 Sample Multiplier: 1

Integration Parameters: autoint1.e
Integrator: ChemStation

Method : C:\msdchem\1\methods\Calibtration plot_Ace.M
Title : autoint1.e

Signal : TIC: 2016.05.05_MADUSHIKA_MM1R3W1.D\data.ms

peak #	R.T. min	first scan	max scan	last scan	PK TY	peak height	corr. area	corr. % max.	% of total
1	11.115	1156	1191	1239	BB 2	169853	7772185	64.72%	39.290%
2	12.648	1426	1450	1489	BB	866123	12009371	100.00%	60.710%

Sum of corrected areas: 19781556

File :C:\msdchem\1\data\Madushika\2016.05.05_MADUSHIKA_MM1C2W1.D
Operator :
Acquired : 5 May 2016 14:15 using AcqMethod Madushika.M
Instrument : UOSJP GCMSD
Sample Name:
Misc Info : ERR
Vial Number: 1

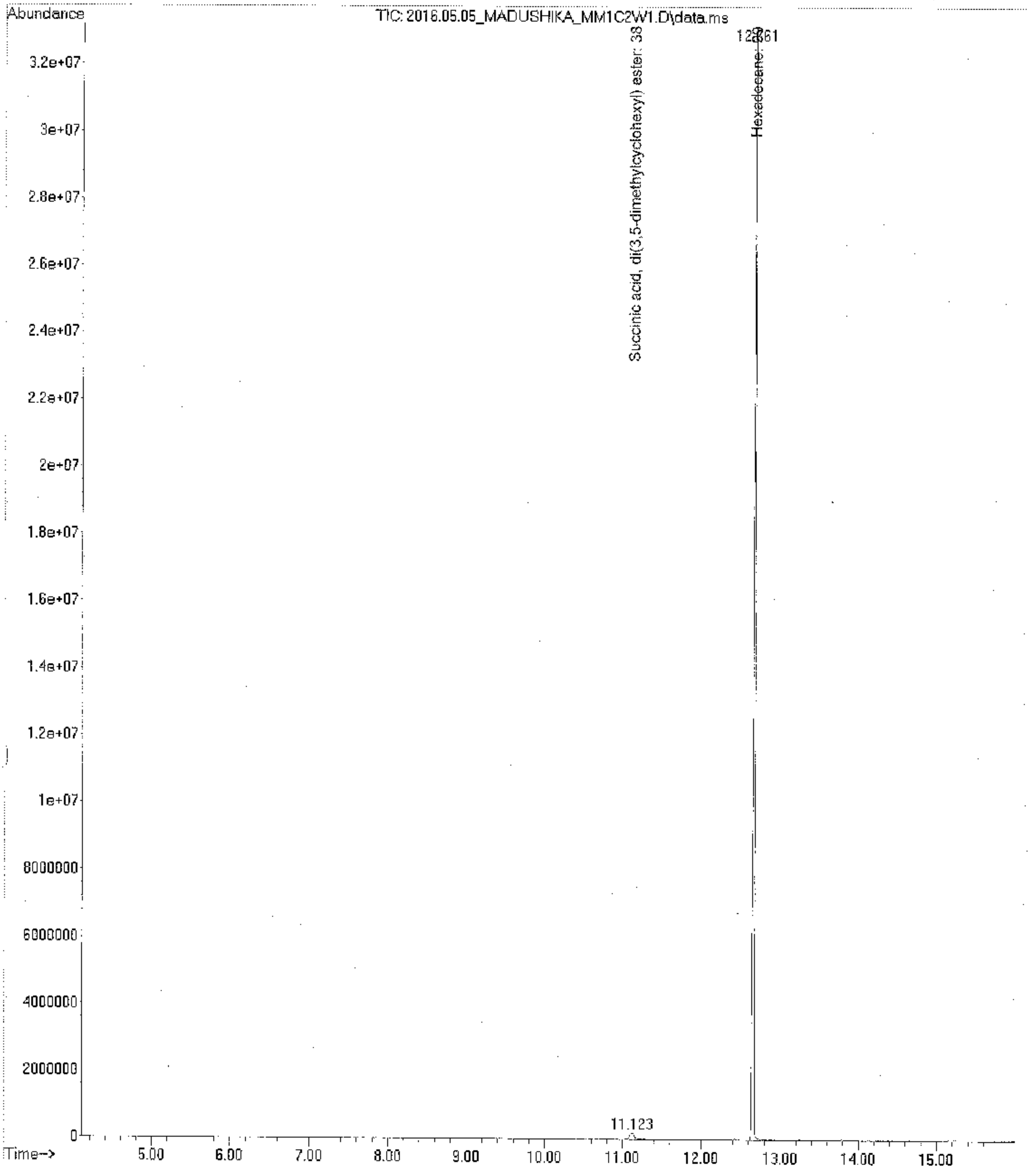

Area Percent Report

Data Path : C:\msdchem\1\data\Madushika\
Data File : 2016.05.05_MADUSHIKA_MM1C2W1.D
Acq On : 5 May 2016 14:15
Operator :
Sample :
Misc :
ALS Vial : 1 Sample Multiplier: 1

Integration Parameters: autoint1.e
Integrator: ChemStation

Method : C:\msdchem\1\methods\Calibtration plot_Ace.M
Title : autoint1.e

Signal : TIC: 2016.05.05_MADUSHIKA_MM1C2W1.D\data.ms

peak #	R.T. min	first scan	max scan	last scan	PK TY	peak height	corr. area	corr. % max.	% of total
1	11.123	1164	1192	1224	BB 3	163083	7573598	1.79%	1.756%
2	12.661	1408	1452	1493	BB	31371039	423681039	100.00%	98.244%

Sum of corrected areas: 431254638

Calibtration plot_Ace.M Thu May 05 14:48:57 2016

File :C:\msdchem\1\data\Madushika\2016.05.05_MADUSHIKA_MM1C3W1.D
Operator :
Acquired : 5 May 2016 14:38 using AcqMethod Madushika.M
Instrument : UOSJP GCMSD
Sample Name:
Misc Info : ERR
Vial Number: 1

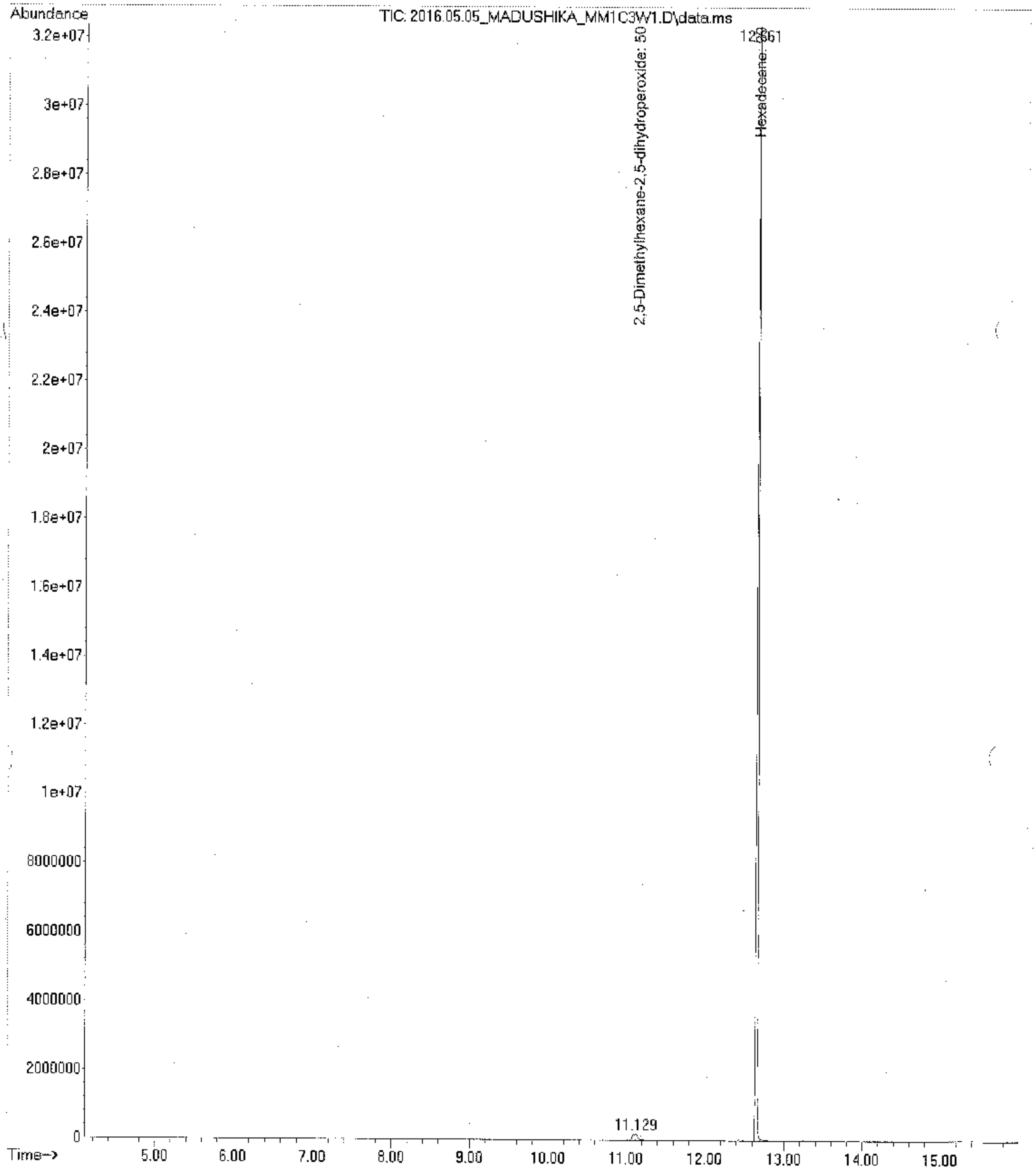

Area Percent Report

Data Path : C:\msdchem\1\data\Madushika\
Data File : 2016.05.05_MADUSHIKA_MM1C3W1.D
Acq On : 5 May 2016 14:38
Operator :
Sample :
Misc :
ALS Vial : 1 Sample Multiplier: 1

Integration Parameters: autoint1.e
Integrator: ChemStation

Method : C:\msdchem\1\methods\Calibtration plot_Ace.M
Title : autoint1.e

Signal : TIC: 2016.05.05_MADUSHIKA_MM1C3W1.D\data.ms

peak #	R.T. min	first scan	max scan	last scan	PK TY	peak height	corr. area	corr. % max.	% of total
1	11.129	1159	1193	1223	BB 2	177412	8541971	2.10%	2.059%
2	12.661	1405	1452	1495	BB	30476237	406225867	100.00%	97.941%

Sum of corrected areas: 414767838

File :C:\msdchem\1\data\Madushika\1_MM9R1W1.D
Operator :
Acquired : 11 May 2016 10:19 using AcqMethod MADUSHIKA.M
Instrument : UOSJP GCMSD
Sample Name: 1_MM9R1W1
Misc Info :
Vial Number: 1

ERR

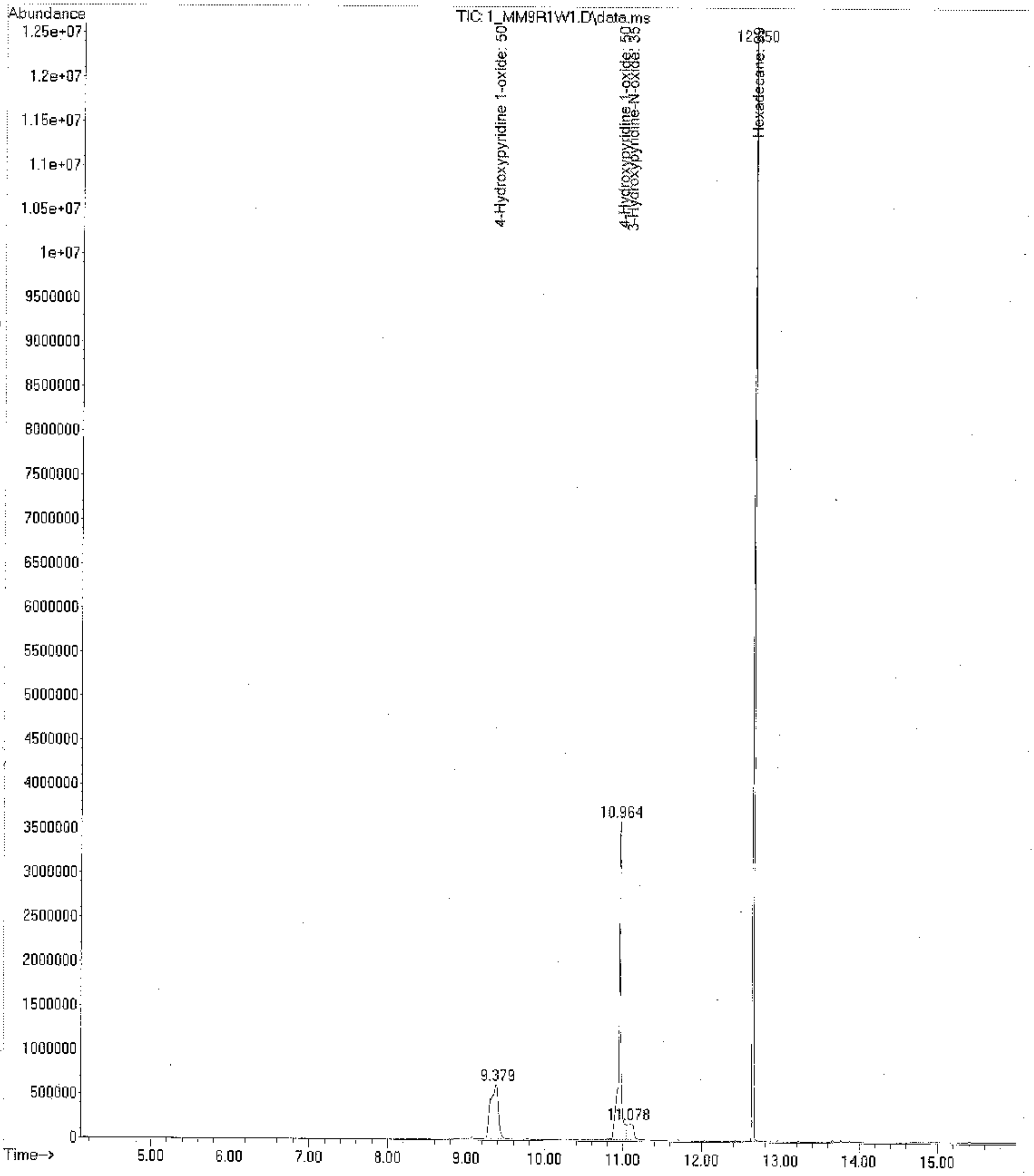

Area Percent Report

Data Path : C:\msdchem\1\data\Madushika\
Data File : 1_MM9R1W1.D
Acq On : 11 May 2016 10:19
Operator :
Sample : 1_MM9R1W1
Misc :
ALS Vial : 1 Sample Multiplier: 1

Integration Parameters: autoint1.e
Integrator: ChemStation

Method : C:\msdchem\1\methods\Calibtration plot_Ace.M
Title : autoint1.e

Signal : TIC: 1_MM9R1W1.D\data.ms

peak #	R.T. min	first scan	max scan	last scan	PK TY	peak height	corr. area	corr. % max.	% of total
1	9.379	857	897	935	BB 2	599019	43821933	33.28%	16.938%
2	10.964	1117	1165	1179	BV	3586592	71846991	54.57%	27.771%
3	11.078	1179	1184	1223	VB 2	185381	11380644	8.64%	4.399%
4	12.650	1405	1451	1473	BB	12094218	131666622	100.00%	50.892%

Sum of corrected areas: 258716190

File :C:\msdchem\1\data\Madushika\2_MM9R2W1.D
Operator :
Acquired : 11 May 2016 10:40 using AcqMethod MADUSHIKA.M
Instrument : UOSJP GCMSD
Sample Name: 2_MM9R2W1
Misc Info : ERR
Vial Number: 2

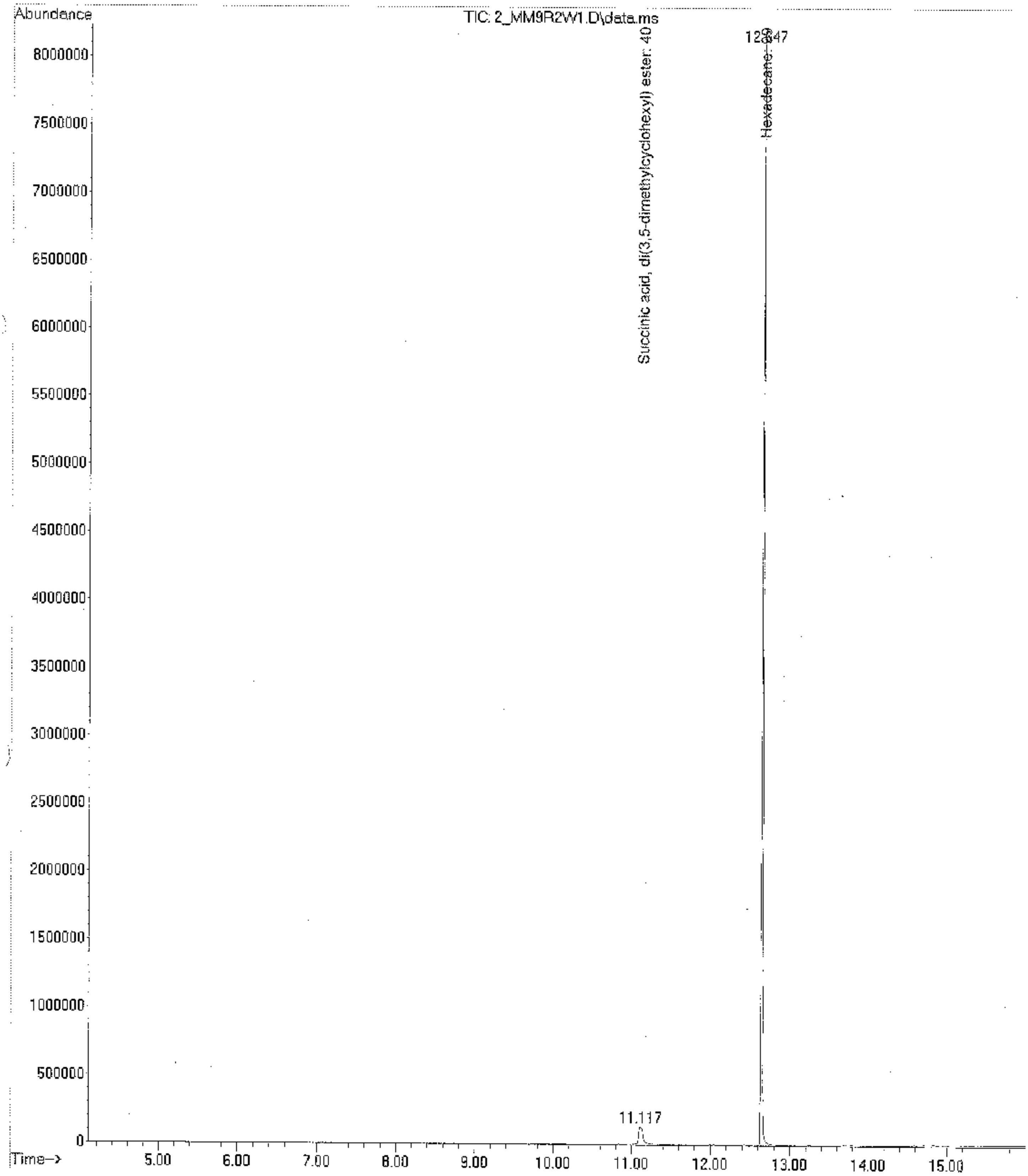

Area Percent Report

Data Path : C:\msdchem\1\data\Madushika\
Data File : 2_MM9R2W1.D
Acq On : 11 May 2016 10:40
Operator :
Sample : 2_MM9R2W1
Misc :
ALS Vial : 2 Sample Multiplier: 1

Integration Parameters: autoint1.e
Integrator: ChemStation

Method : C:\msdchem\1\methods\Calibtration plot_Ace.M
Title : autoint1.e

Signal : TIC: 2_MM9R2W1.D\data.ms

peak #	R.T. min	first scan	max scan	last scan	PK TY	peak height	corr. area	corr. % max.	% of total
1	11.117	1165	1191	1214	BB	121789	4769043	6.07%	5.721%
2	12.647	1430	1450	1481	BB	8005417	78592615	100.00%	94.279%

Sum of corrected areas: 83361658

File :C:\msdchem\1\data\Madushika\3_MM9R3W1.D
Operator :
Acquired : 11 May 2016 11:01 using AcqMethod MADUSHIKA.M
Instrument : UOSJP GCMSD
Sample Name: 3_MM9R3W1
Misc Info : ERR
Vial Number: 3

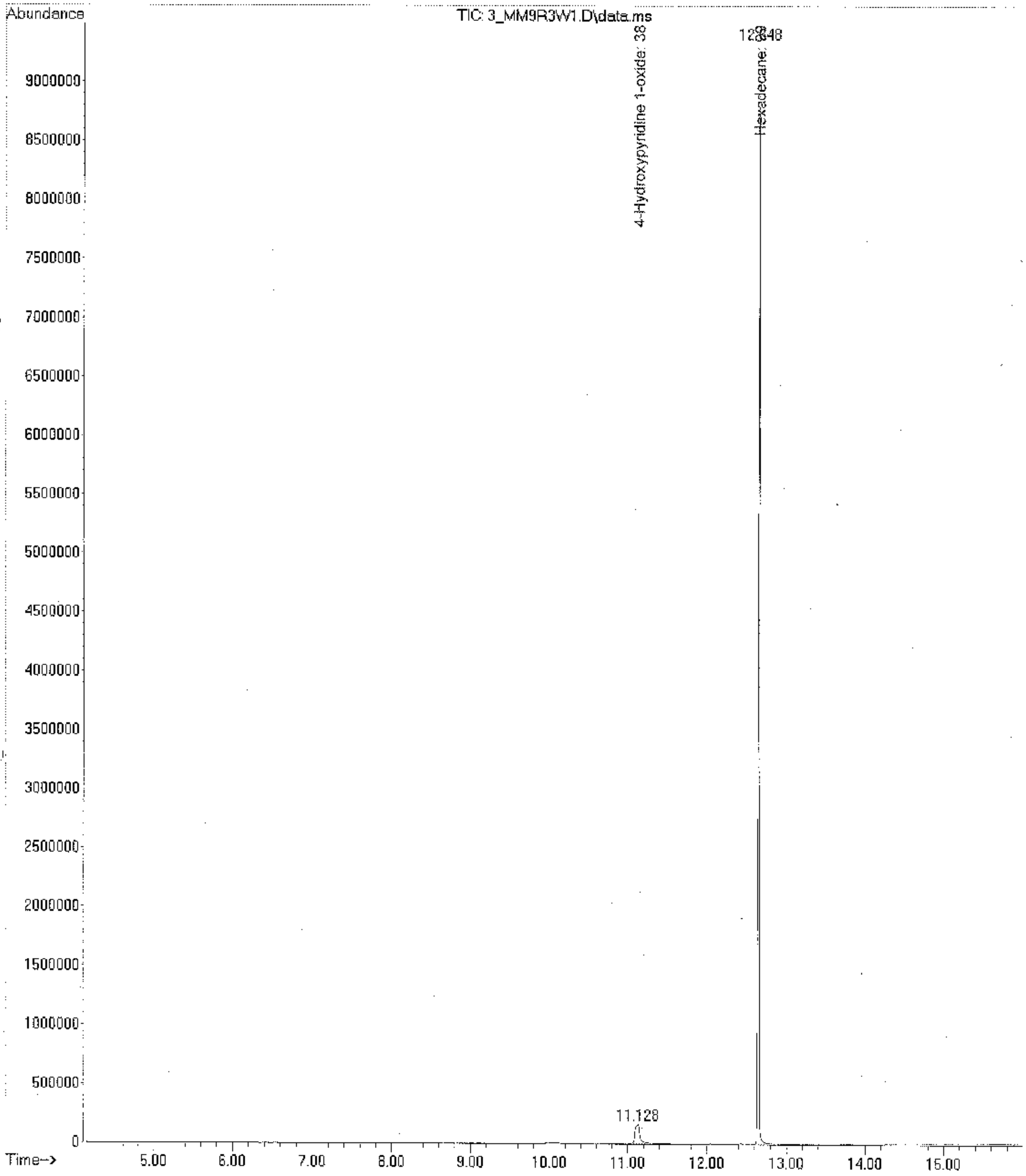

Area Percent Report

Data Path : C:\msdchem\1\data\Madushika\
Data File : 3_MM9R3W1.D
Acq On : 11 May 2016 11:01
Operator :
Sample : 3_MM9R3W1
Misc :
ALS Vial : 3 Sample Multiplier: 1

Integration Parameters: autoint1.e
Integrator: ChemStation

Method : C:\msdchem\1\methods\Calibtration plot_Ace.M
Title : autoint1.e

Signal : TIC: 3_MM9R3W1.D\data.ms

peak #	R.T. min	first scan	max scan	last scan	PK TY	peak height	corr. area	corr. % max.	% of total
1	11.128	1165	1193	1223	BB	158121	6553449	7.44%	6.927%
2	12.648	1434	1450	1479	BB	8851422	88050400	100.00%	93.073%

Sum of corrected areas: 94603849

File :C:\msdchem\1\data\Madushika\4_MM9C1W1.D
Operator :
Acquired : 11 May 2016 11:22 using AcqMethod MADUSHIKA.M
Instrument : UOSJP GCMSD
Sample Name: 4_MM9C1W1
Misc Info : ERR
Vial Number: 4

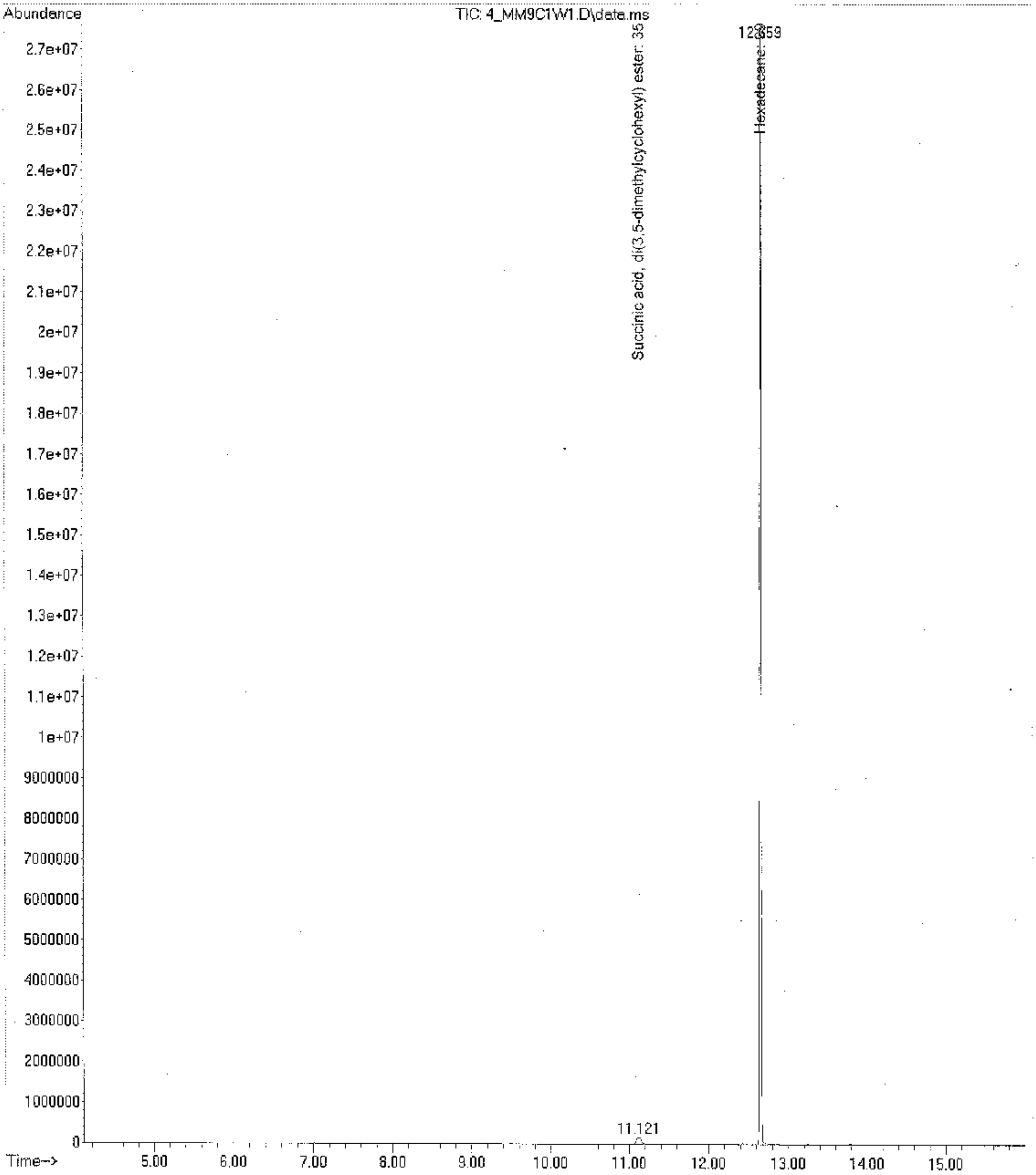

Area Percent Report

Data Path : C:\msdchem\1\data\Madushika\
Data File : 4_MM9C1W1.D
Acq On : 11 May 2016 11:22
Operator :
Sample : 4_MM9C1W1
Misc :
ALS Vial : 4 Sample Multiplier: 1

Integration Parameters: autoint1.e
Integrator: ChemStation

Method : C:\msdchem\1\methods\Calibtration plot_Ace.M
Title : autoint1.e

Signal : TIC: 4_MM9C1W1.D\data.ms

peak #	R.T. min	first scan	max scan	last scan	PK TY	peak height	corr. area	corr. % max.	% of total
1	11.121	1168	1192	1220	BB 2	153072	6780152	2.08%	2.042%
2	12.659	1407	1452	1483	BB	25062144	325297152	100.00%	97.958%

Sum of corrected areas: 332077304

Calibtration plot_Ace.M Fri May 13 10:14:56 2016

File :C:\msdchem\1\data\Madushika\5_MM9C2W1.D
Operator :
Acquired : 11 May 2016 11:43 using AcqMethod MADUSHIKA.M
Instrument : UOSJP GCMSD
Sample Name: 5_MM9C2W1
Misc Info : ERR
Vial Number: 5

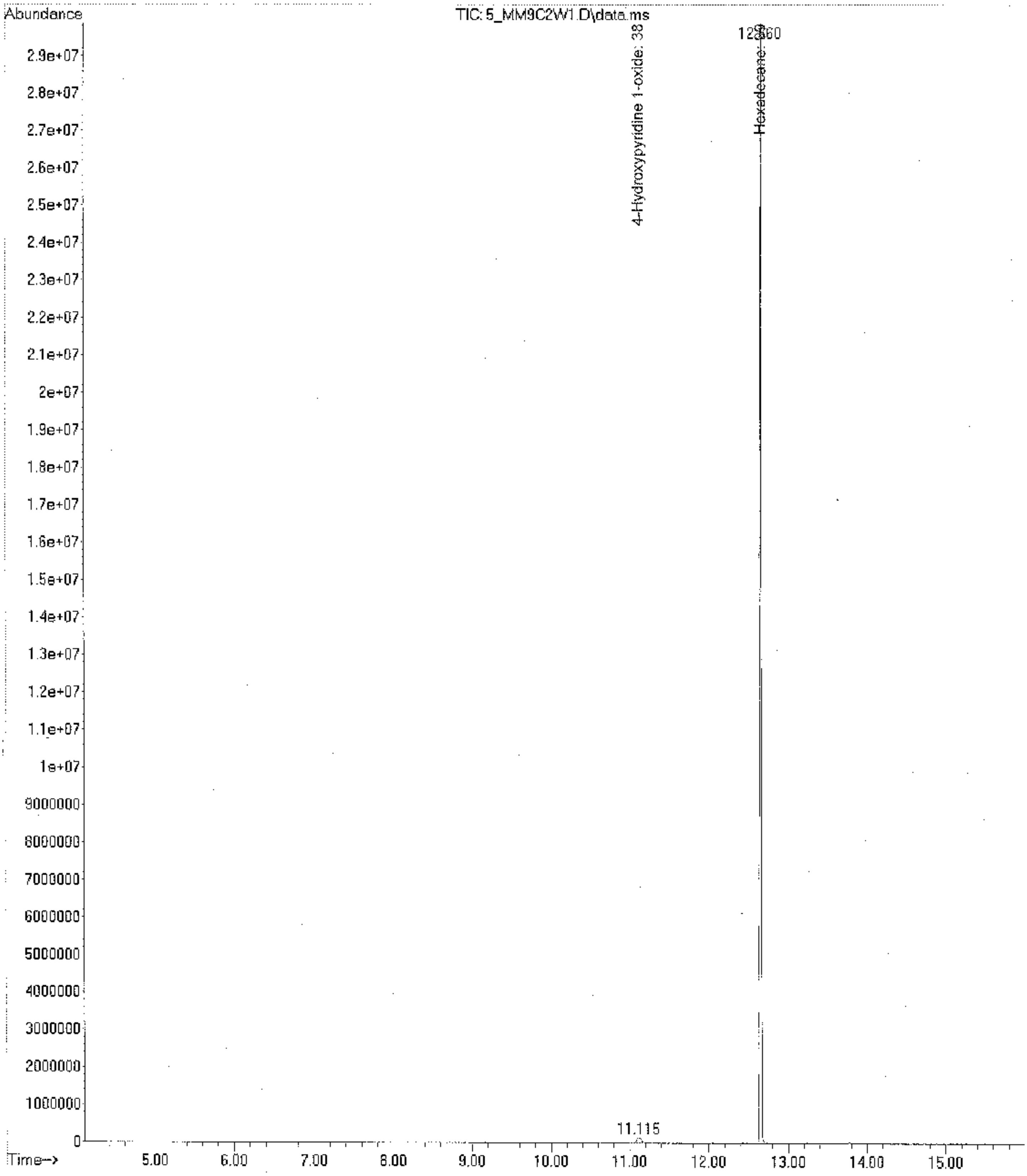

Area Percent Report

Data Path : C:\msdchem\1\data\Madushika\
Data File : 5_MM9C2W1.D
Acq On : 11 May 2016 11:43
Operator :
Sample : 5_MM9C2W1
Misc :
ALS Vial : 5 Sample Multiplier: 1

Integration Parameters: autoint1.e
Integrator: ChemStation

Method : C:\msdchem\1\methods\Calibtration plot_Ace.M
Title : autoint1.e

Signal : TIC: 5_MM9C2W1.D\data.ms

peak #	R.T. min	first scan	max scan	last scan	PK TY	peak height	corr. area	corr. % max.	% of total
1	11.115	1166	1191	1220	BB 4	128240	5613659	1.46%	1.441%
2	12.660	1443	1452	1484	BB	28027788	383958712	100.00%	98.559%

Sum of corrected areas: 389572371

Calibtration plot_Ace.M Fri May 13 10:16:02 2016

File :C:\msdchem\1\data\Madushika\6_MM9C3W1.D
Operator :
Acquired : 11 May 2016 12:04 using AcqMethod MADUSHIKA.M
Instrument : UOSJP GCMSD
Sample Name: 6_MM9C3W1
Misc Info : ERR
Vial Number: 6

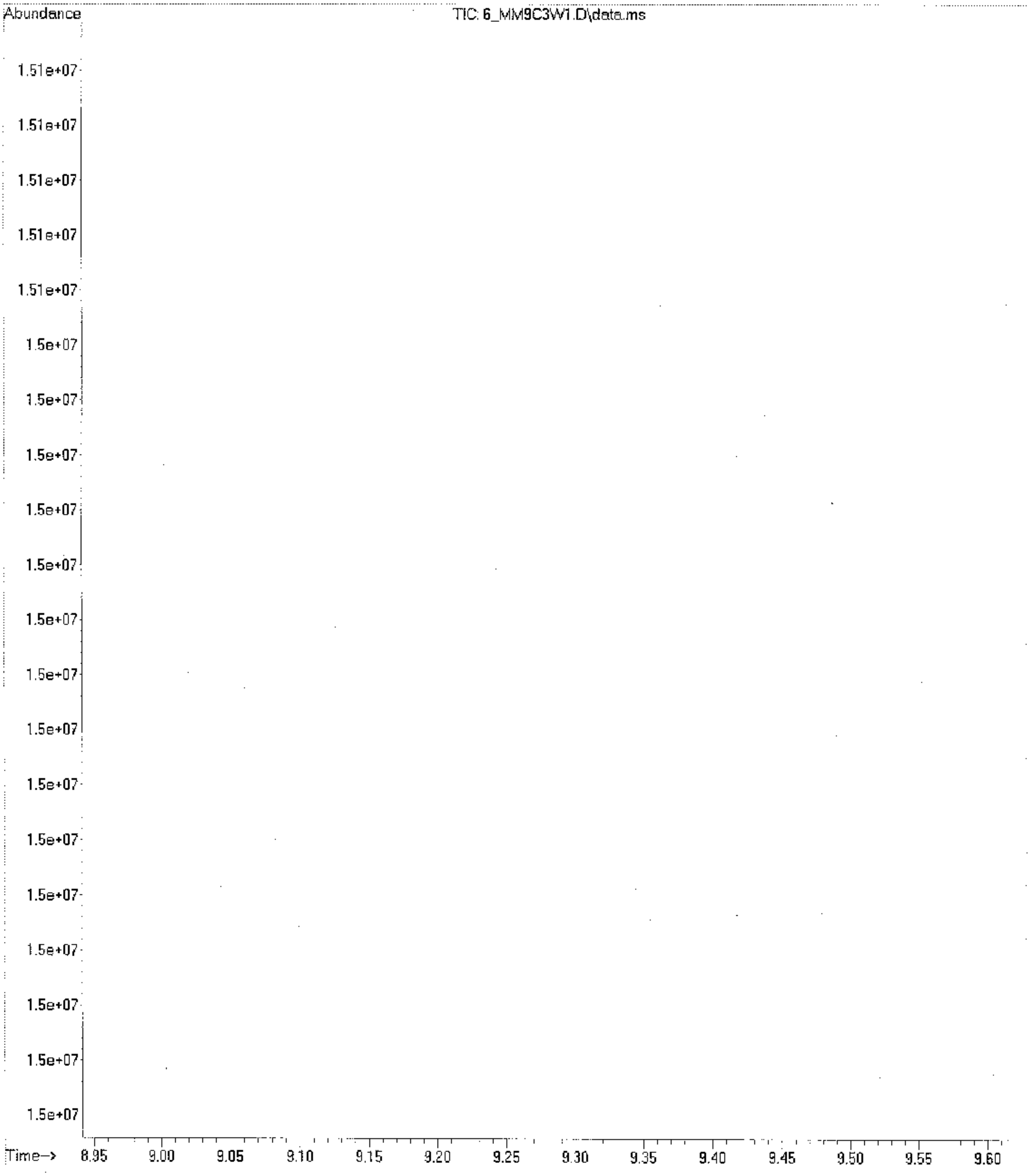

Area Percent Report

Data Path : C:\msdchem\1\data\Madushika\
Data File : 6_MM9C3W1.D
Acq On : 11 May 2016 12:04
Operator :
Sample : 6_MM9C3W1
Misc :
ALS Vial : 6 Sample Multiplier: 1

Integration Parameters: autoint1.e
Integrator: ChemStation

Method : C:\msdchem\1\methods\Calibtration plot_Ace.M
Title : autoint1.e

Signal : TIC: 6_MM9C3W1.D\data.ms

peak #	R.T. min	first scan	max scan	last scan	PK TY	peak height	corr. area	corr. % max.	% of total
1	11.117	1167	1191	1223	BB 2	136449	6180675	1.88%	1.842%
2	12.659	1405	1452	1484	BB	24621656	329326852	100.00%	98.158%

Sum of corrected areas: 335507526

Calibtration plot_Ace.M Fri May 13 10:16:44 2016

File :C:\msdchem\1\data\Madushika\1_MM10R1W1.D
Operator :
Acquired : 13 May 2016 10:09 using AcqMethod MADUSHIKA.M
Instrument : UOSJP GCMSD
Sample Name: 1_MM10R1W1
Misc Info : ERR
Vial Number: 1

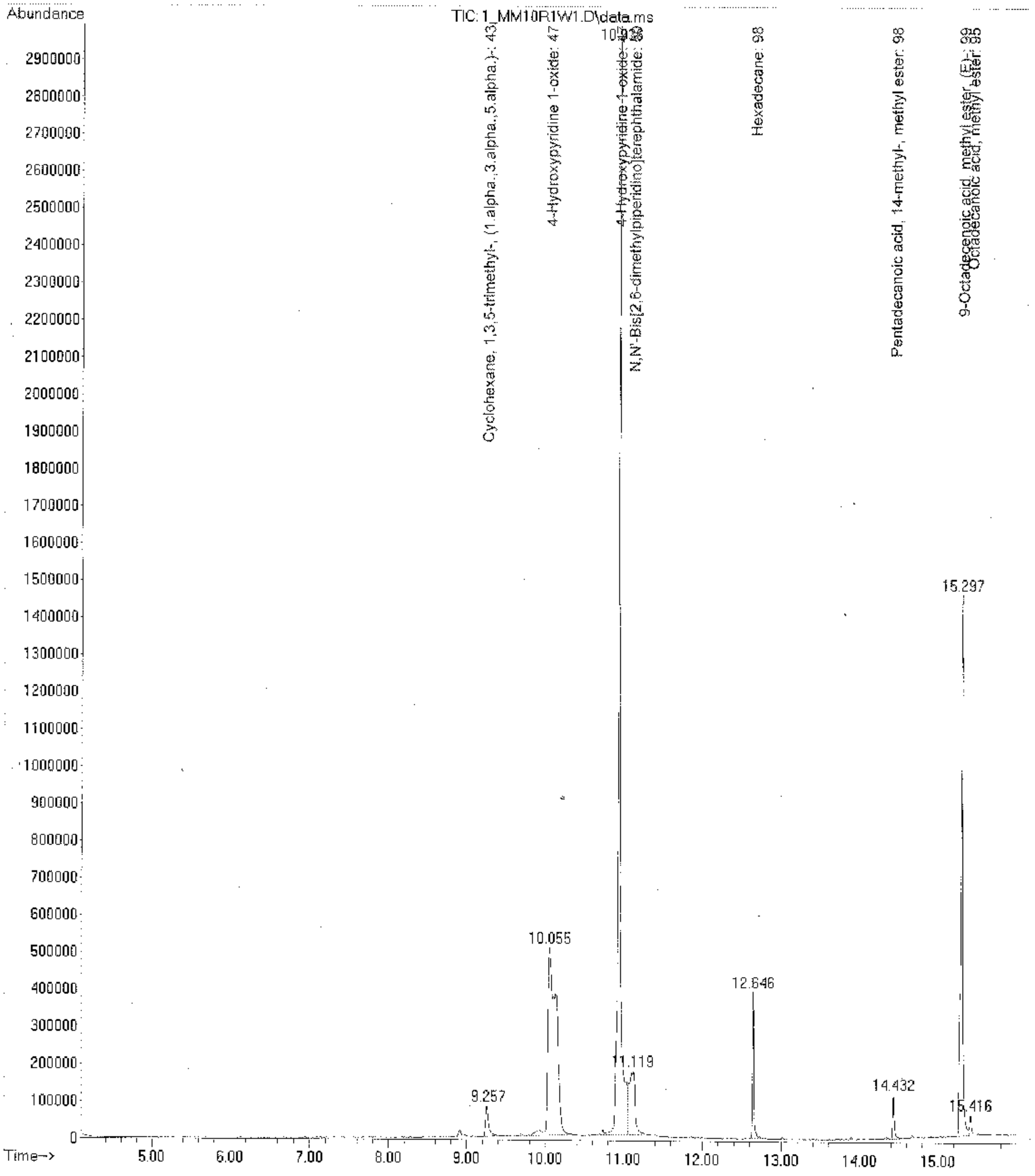

Area Percent Report

Data Path : C:\msdchem\1\data\Madushika\
Data File : 1_MM10R1W1.D
Acq On : 13 May 2016 10:09
Operator :
Sample : 1_MM10R1W1
Misc :
ALS Vial : 1 Sample Multiplier: 1

Integration Parameters: autoint1.e
Integrator: ChemStation

Method : C:\MSDCHEM\1\METHODS\SMART.M
Title : autoint1.e

Signal : TIC: 1_MM10R1W1.D\data.ms

peak #	R.T. min	first scan	max scan	last scan	PK TY	peak height	corr. area	corr. % max.	% of total
1	9.257	867	876	901	BB 2	81487	2251195	4.26%	1.678%
2	10.055	943	1011	1060	BB	483168	36989306	69.98%	27.579%
3	10.936	1110	1160	1180	BV	2855860	52859349	100.00%	39.411%
4	11.119	1180	1191	1220	VB 3	161713	9691725	18.33%	7.226%
5	12.646	1444	1450	1469	BB	354503	4462708	8.44%	3.327%
6	14.432	1727	1753	1773	BB 2	112186	2005618	3.79%	1.495%
7	15.297	1869	1899	1915	BV	1474410	24824851	46.96%	18.509%
8	15.416	1915	1919	1938	VB	53886	1037106	1.96%	0.773%

Sum of corrected areas: 134121858

MART.M Thu May 19 09:33:39 2016

P.T.O.

(Printing mistake)

Area Percent Report

Data Path : C:\msdchem\1\data\Madushika\
Data File : 2_MM10R2W1.D
Acq On : 13 May 2016 10:31
Operator :
Sample : 2_MM10R2W1
Misc :
ALS Vial : 2 Sample Multiplier: 1

Integration Parameters: autoint1.e
Integrator: ChemStation

Method : C:\MSDCHEM\1\METHODS\SMART.M
Title : autoint1.e

Signal : TIC: 2_MM10R2W1.D\data.ms

peak #	R.T. min	first scan	max scan	last scan	PK TY	peak height	corr. area	corr. % max.	% of total
1	11.124	1114	1192	1224	BB 3	168499	8174127	100.00%	58.704%
2	12.646	1444	1450	1469	BB	427677	5750275	70.35%	41.296%

Sum of corrected areas: 13924402

SMART.M Thu May 19 09:40:25 2016

42

```
File        :C:\msdchem\1\data\Madushika\3_MM10R3W1.D
Operator    :
Acquired    : 13 May 2016  10:52      using AcqMethod MADUSHIKA.M
Instrument  :   UOSJP GCMSD
Sample Name: 3_MM10R3W1
Misc Info   :                                              ERR
Vial Number: 3
```

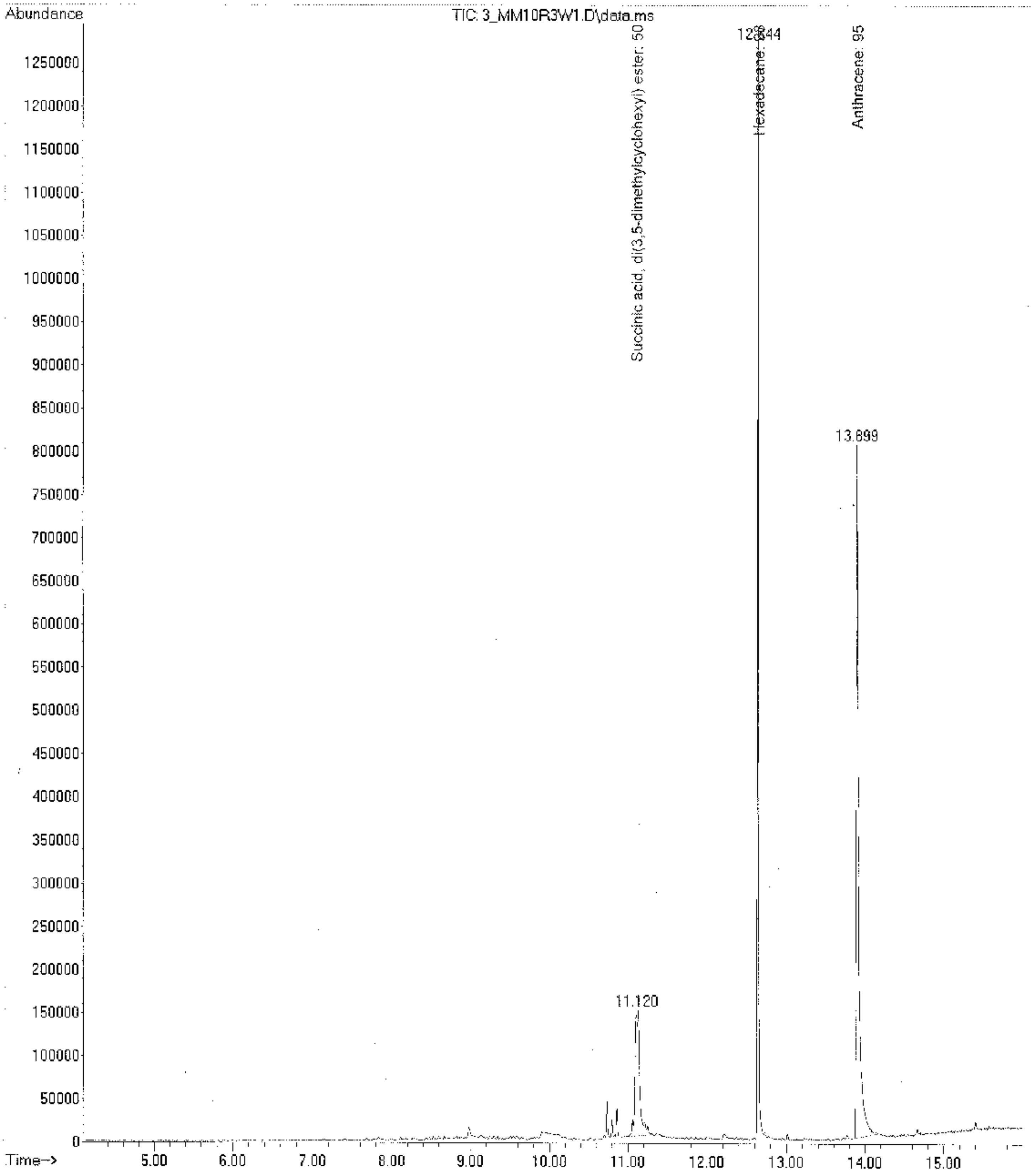

Area Percent Report

Data Path : C:\msdchem\1\data\Madushika\
Data File : 3_MM10R3W1.D
Acq On : 13 May 2016 10:52
Operator :
Sample : 3_MM10R3W1
Misc :
ALS Vial : 3 Sample Multiplier: 1

Integration Parameters: autoint1.e
Integrator: ChemStation

Method : C:\MSDCHEM\1\METHODS\SMART.M
Title : autoint1.e

Signal : TIC: 3_MM10R3W1.D\data.ms

peak #	R.T. min	first scan	max scan	last scan	PK TY	peak height	corr. area	corr. % max.	% of total
1	11.120	1170	1191	1224	BB 2	129660	6167002	37.79%	17.569%
2	12.644	1443	1450	1475	BB	1204960	12615778	77.31%	35.941%
3	13.899	1654	1662	1705	BB	725566	16318855	100.00%	46.490%

Sum of corrected areas: 35101635

SMART.M Thu May 19 09:35:50 2016

File :C:\msdchem\1\data\Madushika\4_MM10C1W1.D
Operator :
Acquired : 13 May 2016 11:13 using AcqMethod MADUSHIKA.M
Instrument : UOSJP GCMSD
Sample Name: 4_MM10C1W1
Misc Info :
Vial Number: 4

ERR

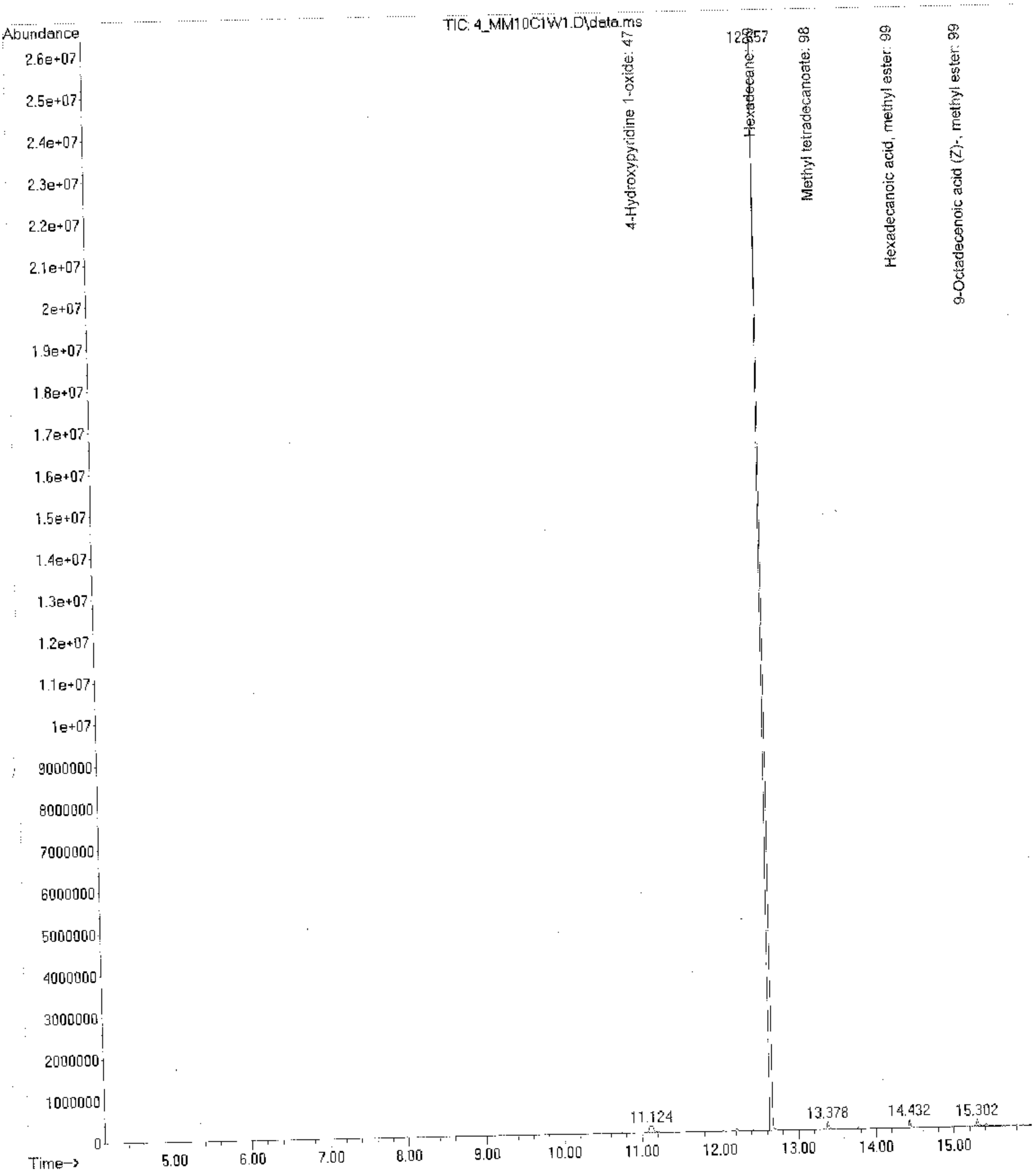

Area Percent Report

Data Path : C:\msdchem\1\data\Madushika\
Data File : 4_MM10C1W1.D
Acq On : 13 May 2016 11:13
Operator :
Sample : 4_MM10C1W1
Misc :
ALS Vial : 4 Sample Multiplier: 1

Integration Parameters: autoint1.e
Integrator: ChemStation

Method : C:\MSDCHEM\1\METHODS\SMART.M
Title : autoint1.e

Signal : TIC: 4_MM10C1W1.D\data.ms

peak #	R.T. min	first scan	max scan	last scan	PK TY	peak height	corr. area	corr. % max.	% of total
1	11.124	1165	1192	1221	BB 3	150610	6611406	2.17%	2.064%
2	12.657	1439	1452	1479	BB	24335899	305060172	100.00%	95.219%
3	13.378	1566	1574	1593	BB	173119	2953665	0.97%	0.922%
4	14.432	1743	1752	1772	BB	185765	2944653	0.97%	0.919%
5	15.302	1876	1900	1914	BV 2	146439	2805841	0.92%	0.876%

Sum of corrected areas: 320375737

File :C:\msdchem\1\data\Madushika\5_MM10C2W1.D
Operator :
Acquired : 13 May 2016 11:34 using AcqMethod MADUSHIKA.M
Instrument : UOSJP GCMSD
Sample Name: 5_MM10C2W1
Misc Info :
Vial Number: 5

ERR

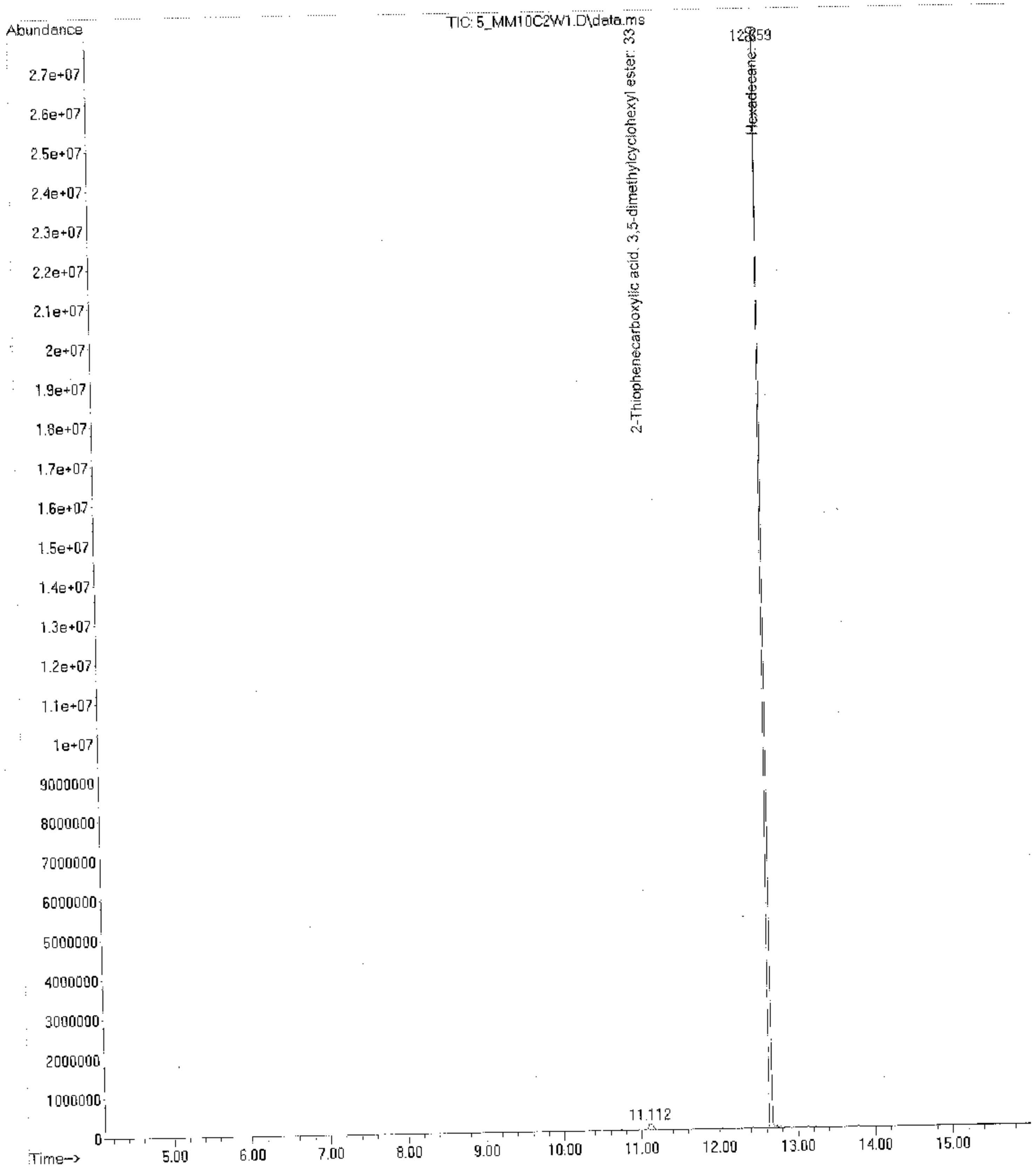

Area Percent Report

Data Path : C:\msdchem\1\data\Madushika\
Data File : 5_MM10C2W1.D
Acq On : 13 May 2016 11:34
Operator :
Sample : 5_MM10C2W1
Misc :
ALS Vial : 5 Sample Multiplier: 1

Integration Parameters: autoint1.e
Integrator: ChemStation

Method : C:\MSDCHEM\1\METHODS\SMART.M
Title : autoint1.e

Signal : TIC: 5_MM10C2W1.D\data.ms

peak #	R.T. min	first scan	max scan	last scan	PK TY	peak height	corr. area	corr. % max.	% of total
1	11.112	1166	1190	1220	BB	145938	6370194	1.94%	1.906%
2	12.659	1443	1452	1469	BV	25533244	327778543	100.00%	98.094%

Sum of corrected areas: 334148737

SMART.M Thu May 19 09:37:05 2016

File :C:\msdchem\1\data\Madushika\6_MM10C3W1.D
Operator :
Acquired : 13 May 2016 11:55 using AcqMethod MADUSHIKA.M
Instrument : UOSJP GCMSD
Sample Name: 6_MM10C3W1
Misc Info :
Vial Number: 6

ERR

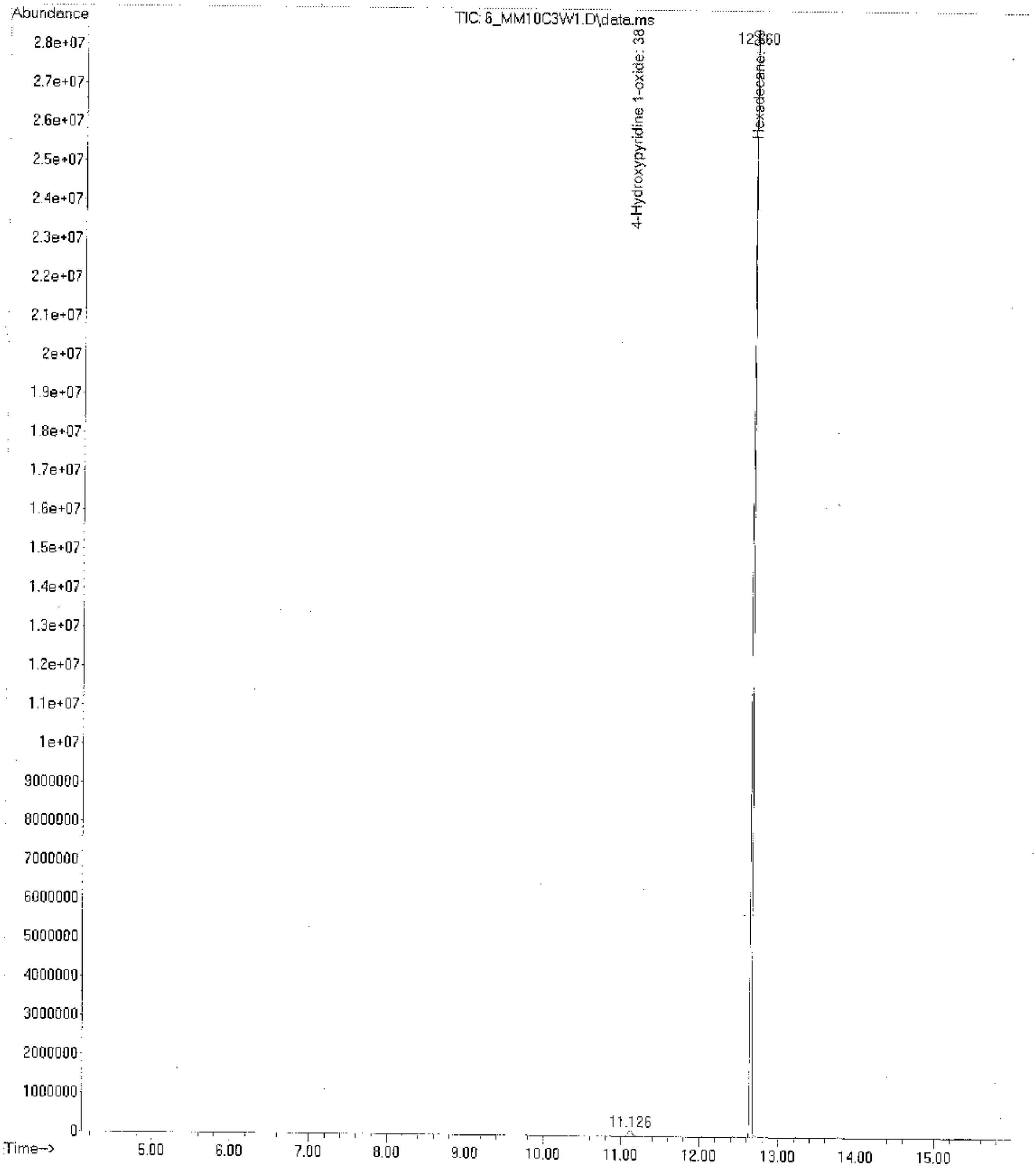

Area Percent Report

Data Path : C:\msdchem\1\data\Madushika\
Data File : 6_MM10C3W1.D
Acq On : 13 May 2016 11:55
Operator :
Sample : 6_MM10C3W1
Misc :
ALS Vial : 6 Sample Multiplier: 1

Integration Parameters: autoint1.e
Integrator: ChemStation

Method : C:\MSDCHEM\1\METHODS\SMART.M
Title : autoint1.e

Signal : TIC: 6_MM10C3W1.D\data.ms

peak #	R.T. min	first scan	max scan	last scan	PK TY	peak height	corr. area	corr. % max.	% of total
1	11.126	1165	1192	1222	BB 2	128652	5801117	1.53%	1.504%
2	12.660	1443	1452	1481	BB	26362274	379891099	100.00%	98.496%

Sum of corrected areas: 385692217

SMART.M Thu May 19 09:38:53 2016

MM1FungiR1 46

```
File        :C:\msdchem\1\data\Madushika\1_CR1W1.D
Operator    :
Acquired    : 10 Jun 2016   9:52    using AcqMethod MADUSHIKA.M
Instrument  :   UOSJP GCMSD
Sample Name: 1_CR1W1
Misc Info   :                                        ERR
Vial Number: 1
```

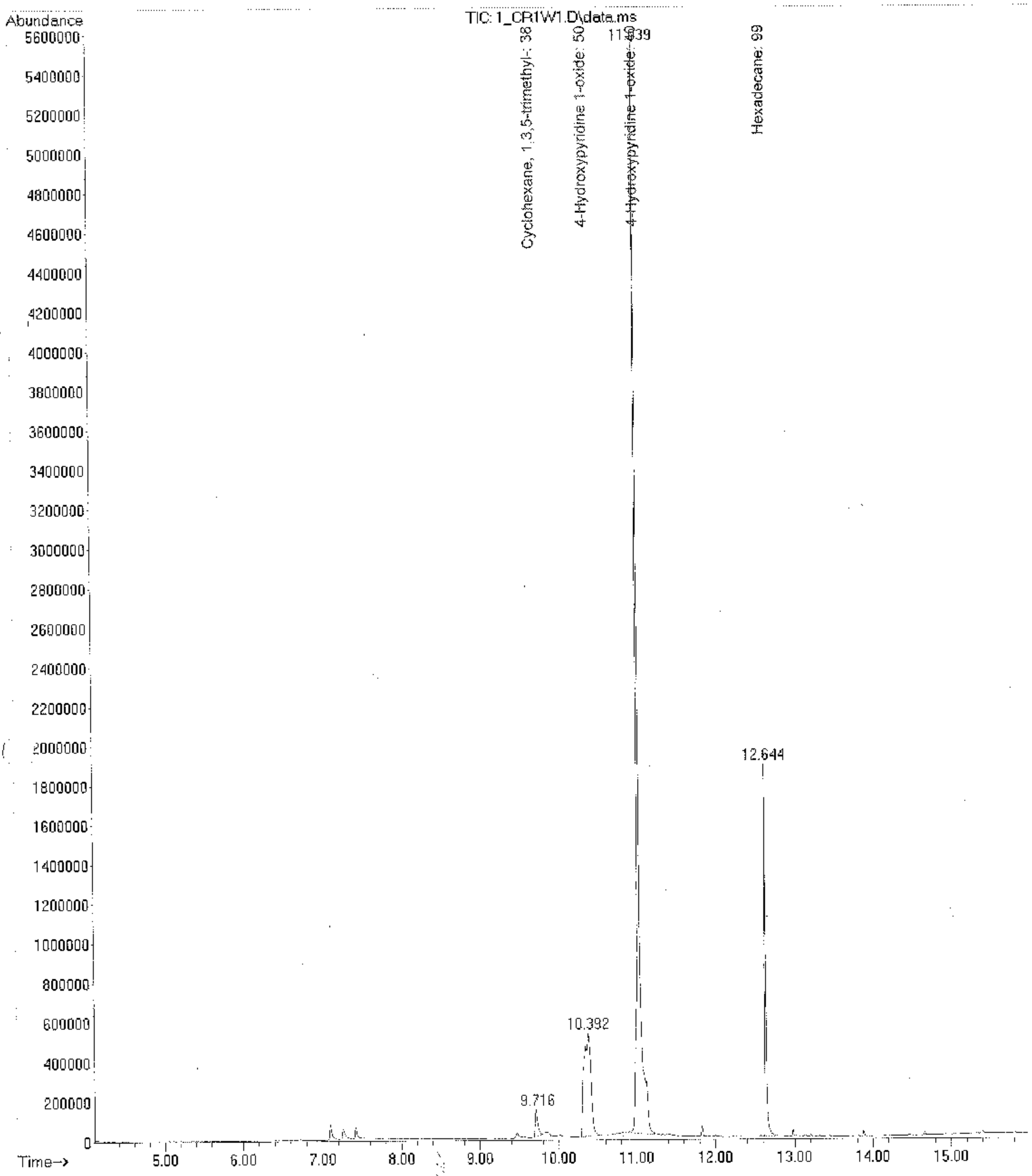

Area Percent Report

Data Path : C:\msdchem\1\data\Madushika\
Data File : 1_CR1W1.D
Acq On : 10 Jun 2016 9:52
Operator :
Sample : 1_CR1W1
Misc :
ALS Vial : 1 Sample Multiplier: 1

Integration Parameters: autoint1.e
Integrator: ChemStation

Method : C:\msdchem\1\methods\Calibtration plot_Ace.M
Title : autoint1.e

Signal : TIC: 1_CR1W1.D\data.ms

peak #	R.T. min	first scan	max scan	last scan	PK TY	peak height	corr. area	corr. % max.	% of total
1	9.716	945	954	965	BV 2	129567	3060605	3.07%	1.980%
2	10.392	1046	1068	1090	BB	520077	32490914	32.63%	21.018%
3	11.039	1144	1178	1224	BB	5124962	99588600	100.00%	64.422%
4	12.644	1441	1450	1475	BB	1742704	19448052	19.53%	12.581%

Sum of corrected areas: 154588170

alibtration plot_Ace.M Mon Jun 13 09:56:33 2016

MM|Fungi R2 53.

File :C:\msdchem\1\data\Madushika\2_CR2W1.D
Operator :
Acquired : 10 Jun 2016 10:13 using AcqMethod MADUSHIKA.M
Instrument : UOSJP GCMSD
Sample Name: 2_CR2W1
Misc Info : ERR
Vial Number: 2

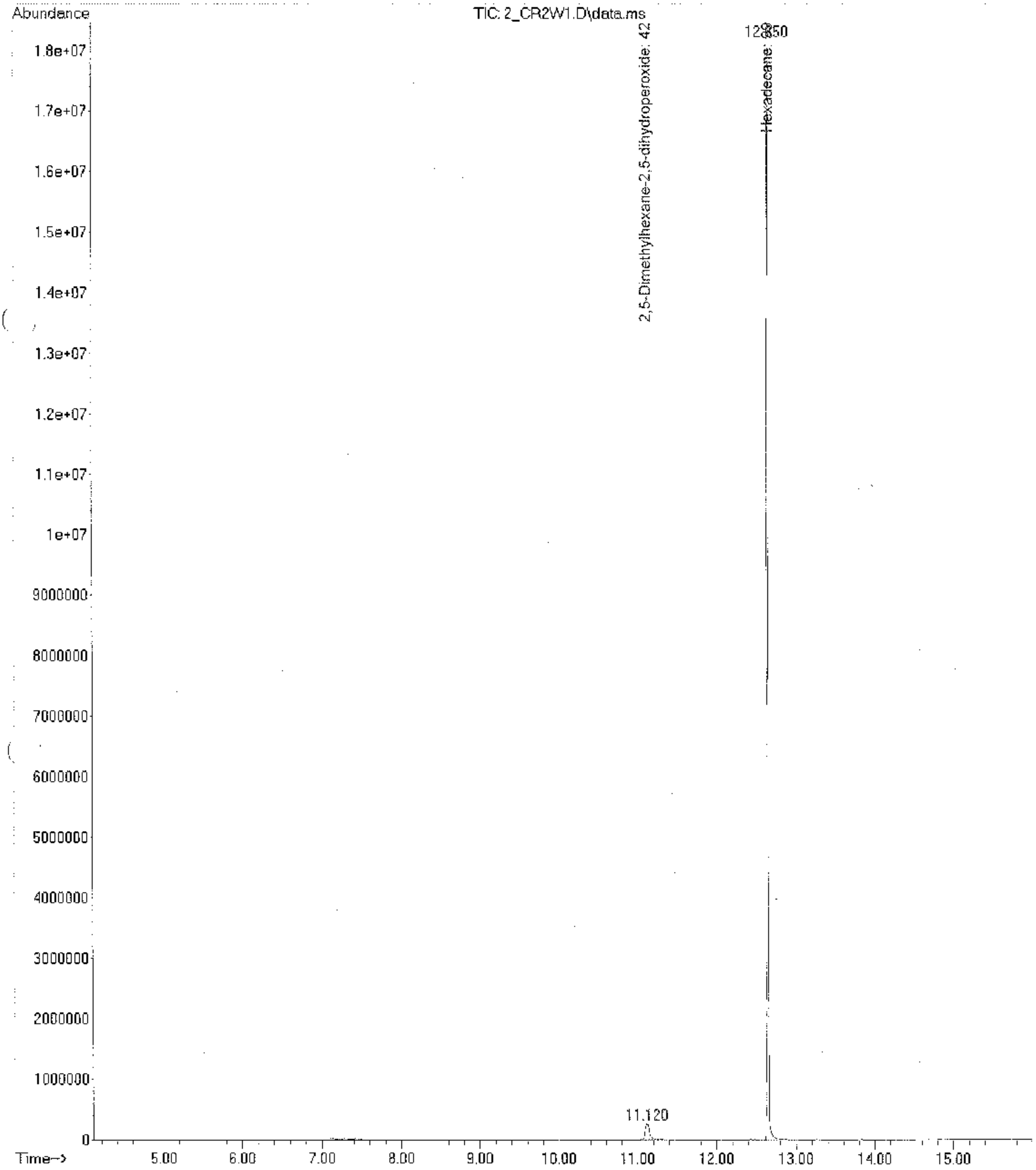

Area Percent Report

Data Path : C:\msdchem\1\data\Madushika\
Data File : 2_CR2W1.D
Acq On : 10 Jun 2016 10:13
Operator :
Sample : 2_CR2W1
Misc :
ALS Vial : 2 Sample Multiplier: 1

Integration Parameters: autoint1.e
Integrator: ChemStation

Method : C:\msdchem\1\methods\Calibtration plot_Ace.M
Title : autoint1.e

Signal : TIC: 2_CR2W1.D\data.ms

peak #	R.T. min	first scan	max scan	last scan	PK TY	peak height	corr. area	corr. % max.	% of total
1	11.120	1169	1191	1217	BB 4	242232	9898849	4.59%	4.388%
2	12.650	1433	1451	1484	BB	18008167	215666831	100.00%	95.612%

Sum of corrected areas: 225565680

libtration plot_Ace.M Mon Jun 13 09:58:16 2016

MMIfungiR3 34

C = fungi of MMI

File :C:\msdchem\1\data\Madushika\3_CR3W1.D
Operator :
Acquired : 10 Jun 2016 10:34 using AcqMethod MADUSHIKA.M
Instrument : UOSJP GCMSD
Sample Name: 3_CR3W1
Misc Info : ERR
Vial Number: 3

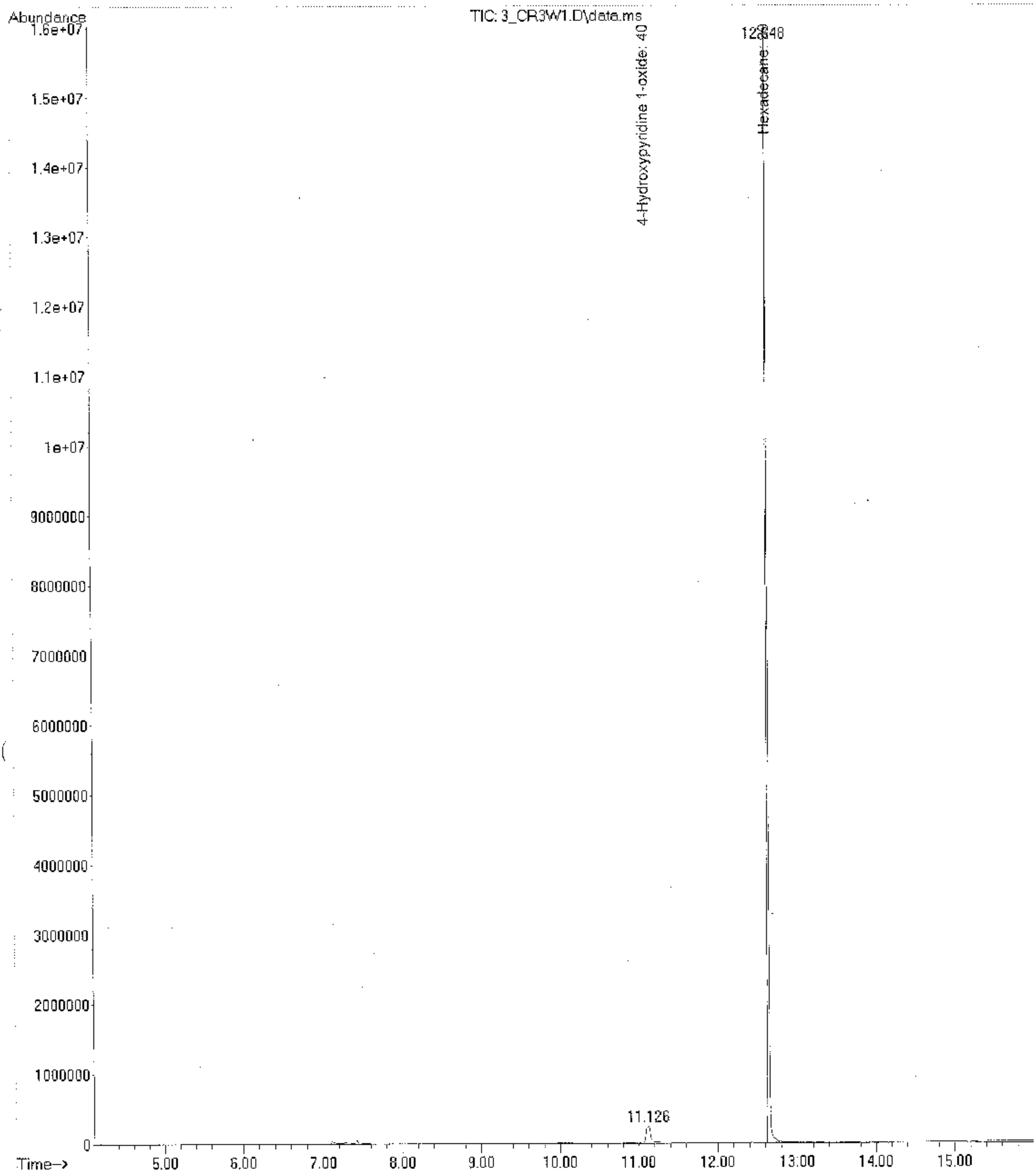

Area Percent Report

Data Path : C:\msdchem\1\data\Madushika\
Data File : 3_CR3W1.D
Acq On : 10 Jun 2016 10:34
Operator :
Sample : 3_CR3W1
Misc :
ALS Vial : 3 Sample Multiplier: 1

Integration Parameters: autoint1.e
Integrator: ChemStation

Method : C:\msdchem\1\methods\Calibtration plot_Ace.M
Title : autoint1.e

Signal : TIC: 3_CR3W1.D\data.ms

eak #	R.T. min	first scan	max scan	last scan	PK TY	peak height	corr. area	corr. % max.	% of total
1	11.126	1160	1192	1218	BB	218724	8923751	5.40%	5.123%
2	12.648	1440	1450	1483	BB	15222188	165266112	100.00%	94.877%

Sum of corrected areas: 174189863

55.
MM1 Fungi Control

File :C:\msdchem\1\data\Madushika\4_CC1W1.D
Operator :
Acquired : 10 Jun 2016 10:56 using AcqMethod MADUSHIKA.M
Instrument : UOSJP GCMSD
Sample Name: 4_CC1W1
Misc Info : ERR
Vial Number: 4

CC1 ⇒ control 1 of MM1 fungi

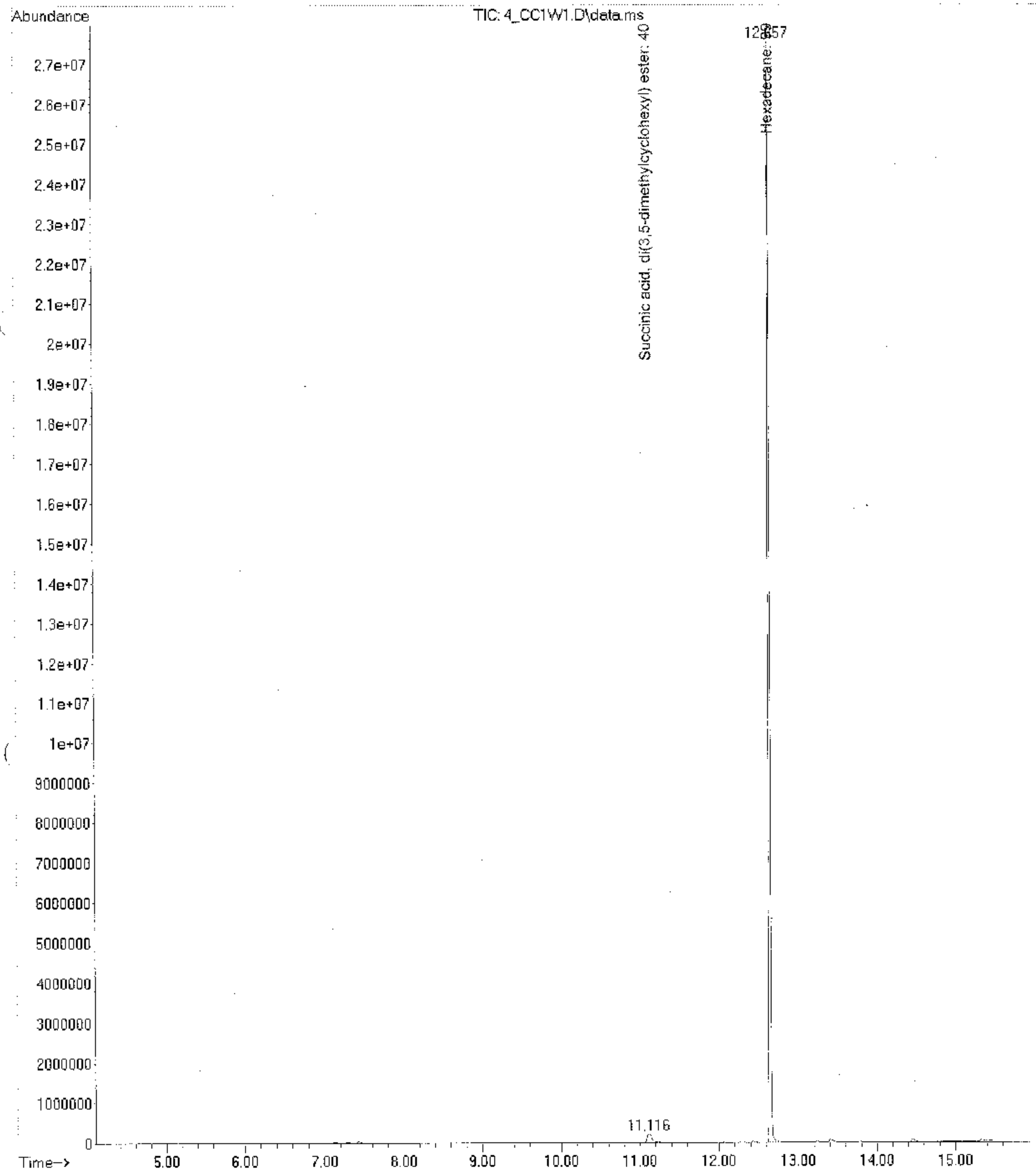

Area Percent Report

Data Path : C:\msdchem\1\data\Madushika\
Data File : 4_CC1W1.D
Acq On : 10 Jun 2016 10:56
Operator :
Sample : 4_CC1W1
Misc :
ALS Vial : 4 Sample Multiplier: 1

Integration Parameters: autoint1.e
Integrator: ChemStation

Method : C:\msdchem\1\methods\Calibtration plot_Ace.M
Title : autoint1.e

Signal : TIC: 4_CC1W1.D\data.ms

eak #	R.T. min	first scan	max scan	last scan	PK TY	peak height	corr. area	corr. % max.	% of total
1	11.116	1161	1191	1217	BB 3	184670	8044345	2.04%	2.003%
2	12.657	1407	1452	1481	BB	26051900	393472335	100.00%	97.997%

Sum of corrected areas: 401516680

56
MMIFungi Cont

File :C:\msdchem\1\data\Madushika\5_CC2W1.D
Operator :
Acquired : 10 Jun 2016 11:17 using AcqMethod MADUSHIKA.M
Instrument : UOSJP GCMSD
Sample Name: 5_CC2W1
Misc Info :
Vial Number: 5

ERR

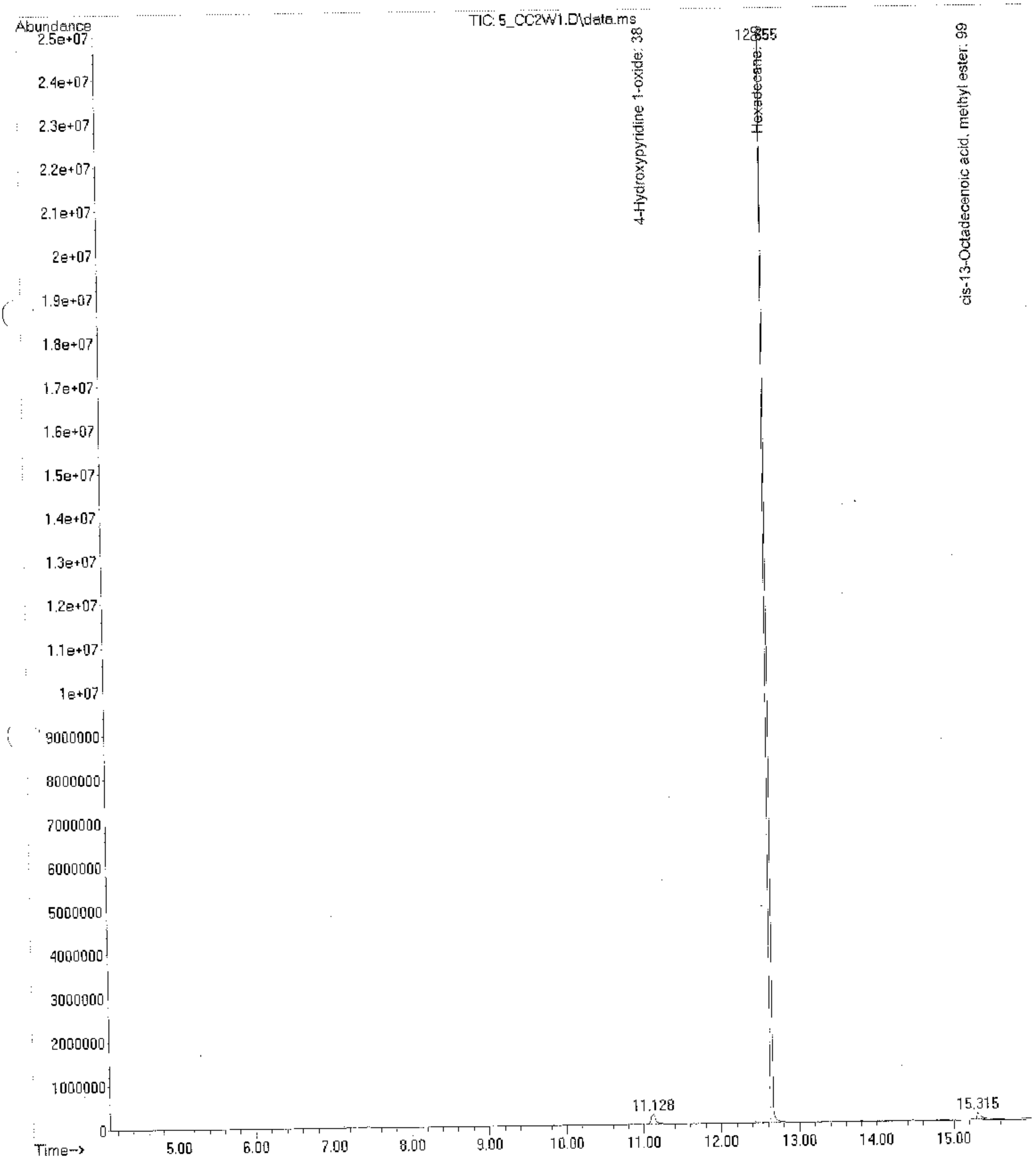

Area Percent Report

Data Path : C:\msdchem\1\data\Madushika\
Data File : 5_CC2W1.D
Acq On : 10 Jun 2016 11:17
Operator :
Sample : 5_CC2W1
Misc :
ALS Vial : 5 Sample Multiplier: 1

Integration Parameters: autoint1.e
Integrator: ChemStation

Method : C:\msdchem\1\methods\Calibtration plot_Ace.M
Title : autoint1.e

Signal : TIC: 5_CC2W1.D\data.ms

eak #	R.T. min	first scan	max scan	last scan	PK TY	peak height	corr. area	corr. % max.	% of total
1	11.128	1164	1193	1218	BB	209905	8276886	2.54%	2.442%
2	12.655	1408	1451	1484	BB	23101541	325934944	100.00%	96.152%
3	15.315	1874	1902	1931	BB 2	133918	4766154	1.46%	1.406%

Sum of corrected areas: 338977984

libtration plot_Ace.M Mon Jun 13 10:01:49 2016

57.

MMI Fungi Control

File :C:\msdchem\1\data\Madushika\6_CC3W1.D
Operator :
Acquired : 10 Jun 2016 11:38 using AcqMethod MADUSHIKA.M
Instrument : UOSJP GCMSD
Sample Name: 6_CC3W1
Misc Info :
Vial Number: 6

ERR

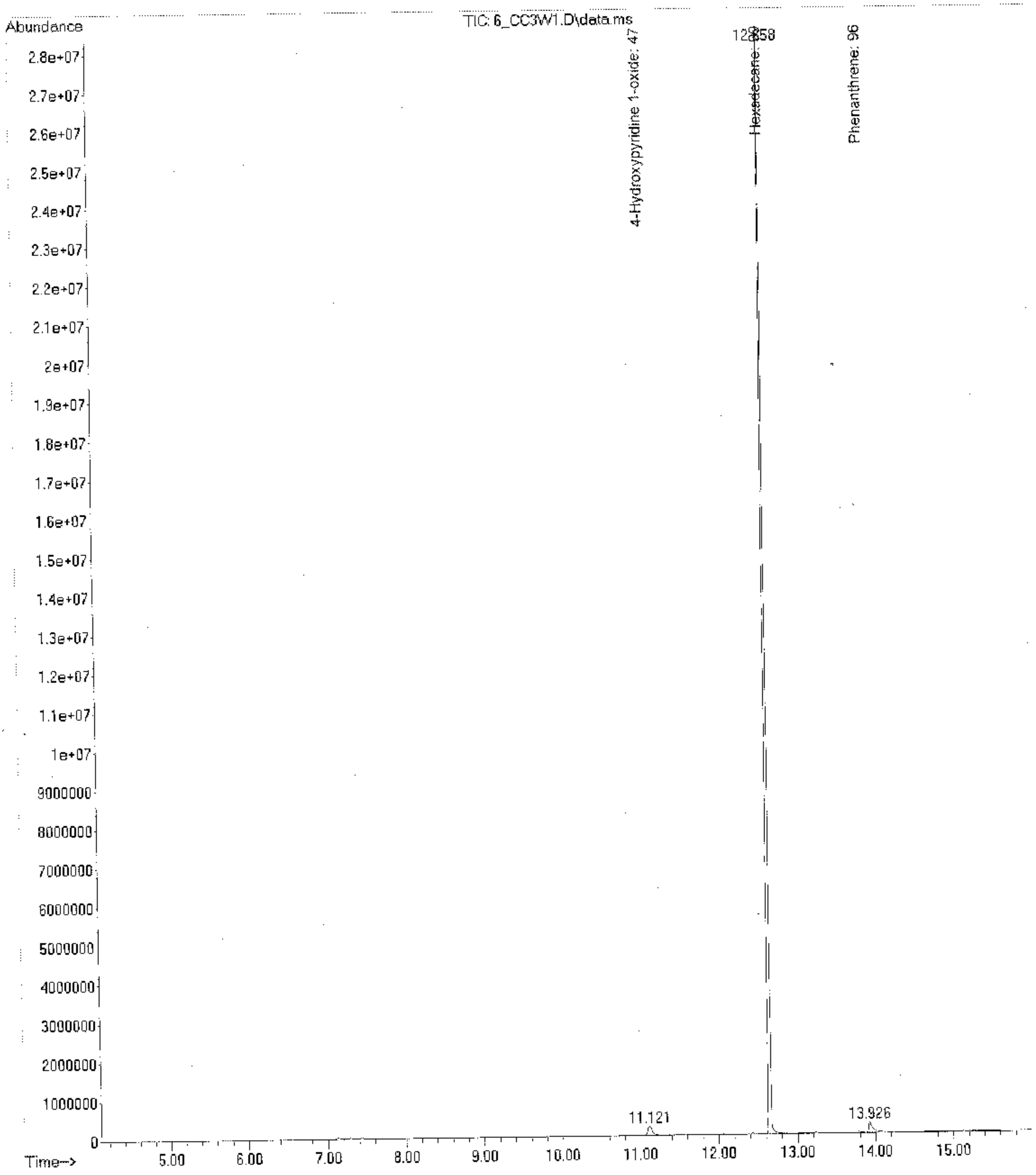

Area Percent Report

Data Path : C:\msdchem\1\data\Madushika\
Data File : 6_CC3W1.D
Acq On : 10 Jun 2016 11:38
Operator :
Sample : 6_CC3W1
Misc :
ALS Vial : 6 Sample Multiplier: 1

Integration Parameters: autoint1.e
Integrator: ChemStation

Method : C:\msdchem\1\methods\Calibtration plot_Ace.M
Title : autoint1.e

Signal : TIC: 6_CC3W1.D\data.ms

peak #	R.T. min	first scan	max scan	last scan	PK TY	peak height	corr. area	corr. % max.	% of total
1	11.121	1163	1192	1217	BB 3	217680	8560779	1.98%	1.896%
2	12.658	1406	1452	1484	BB	26678762	433449019	100.00%	96.008%
3	13.926	1656	1667	1703	BB	244906	9462345	2.18%	2.096%

Sum of corrected areas: 451472143

MMIBacR1

File :C:\msdchem\1\data\Madushika\1_BR1W1.D
Operator :
Acquired : 10 Jun 2016 12:08 using AcqMethod MADUSHIKA.M
Instrument : UOSJP GCMSD
Sample Name: 1_BR1W1
Misc Info : ERR
Vial Number: 1

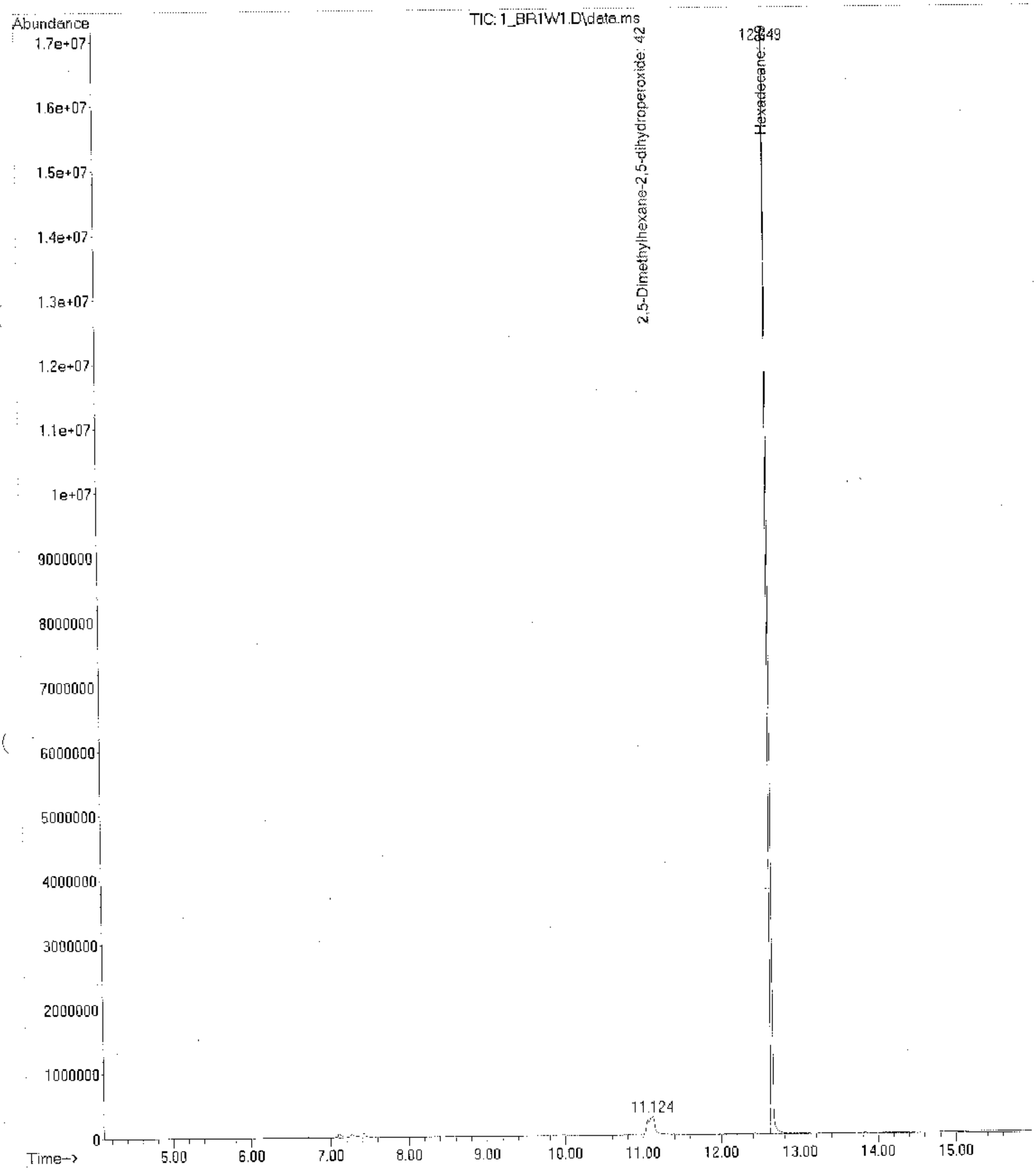

Area Percent Report

Data Path : C:\msdchem\1\data\Madushika\
Data File : 1_BR1W1.D
Acq On : 10 Jun 2016 12:08
Operator :
Sample : 1_BR1W1
Misc :
ALS Vial : 1 Sample Multiplier: 1

Integration Parameters: autoint1.e
Integrator: ChemStation

Method : C:\msdchem\1\methods\Calibtration plot_Ace.M
Title : autoint1.e

Signal : TIC: 1_BR1W1.D\data.ms

peak #	R.T. min	first scan	max scan	last scan	PK TY	peak height	corr. area	corr. % max.	% of total
1	11.124	1153	1192	1222	BB	269796	16901064	8.69%	7.998%
2	12.649	1431	1450	1484	BB	16502119	194408494	100.00%	92.002%

Sum of corrected areas: 211309559

alibtration plot_Ace.M Mon Jun 13 09:57:14 2016

MMI Bac R 2

File :C:\msdchem\1\data\Madushika\2_BR2W1.D
Operator :
Acquired : 10 Jun 2016 12:29 using AcqMethod MADUSHIKA.M
Instrument : UOSJP GCMSD
Sample Name: 2_BR2W1
Misc Info : ERR
Vial Number: 2

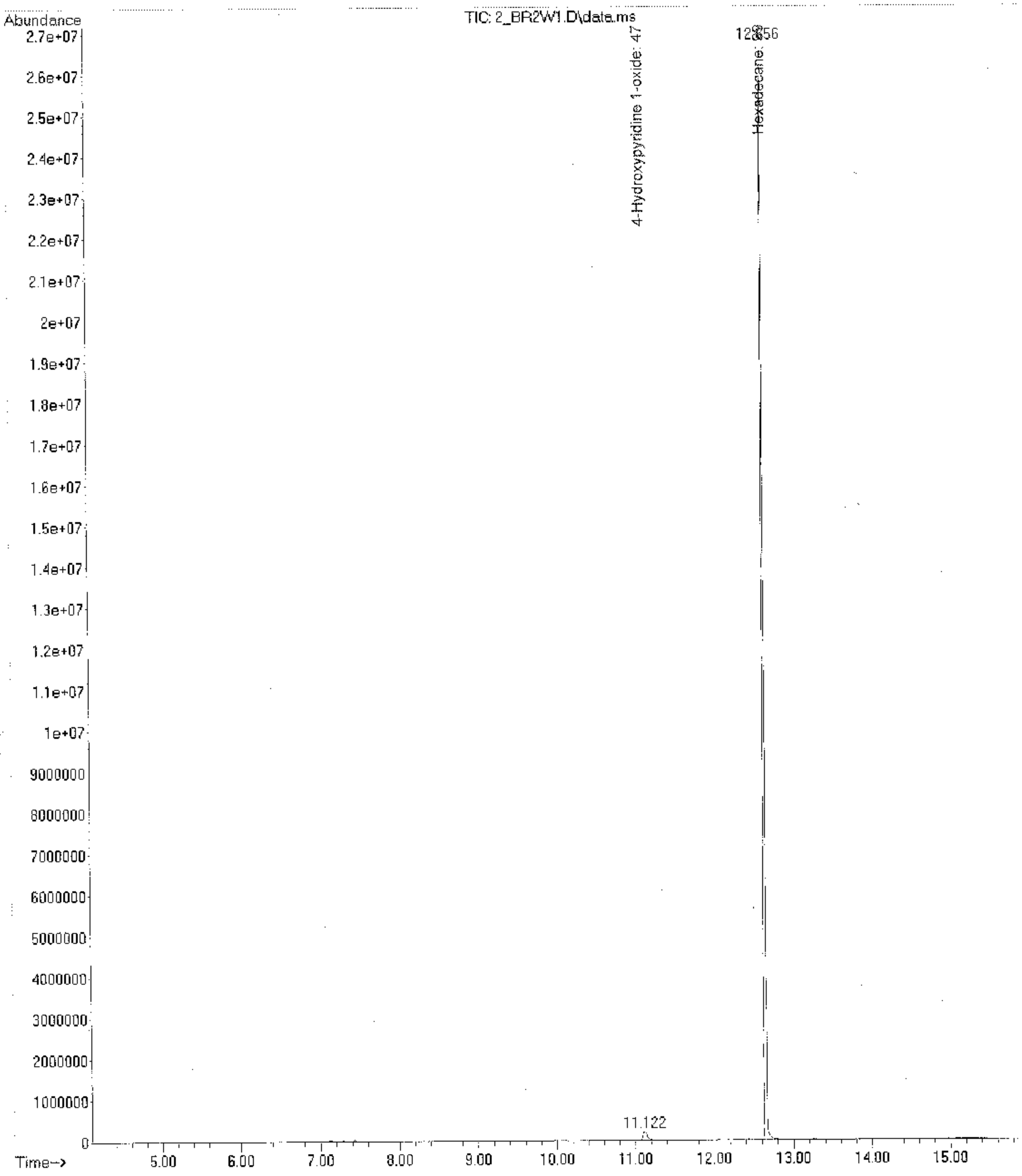

Area Percent Report

Data Path : C:\msdchem\1\data\Madushika\
Data File : 2_BR2W1.D
Acq On : 10 Jun 2016 12:29
Operator :
Sample : 2_BR2W1
Misc :
ALS Vial : 2 Sample Multiplier: 1

Integration Parameters: autoint1.e
Integrator: ChemStation

Method : C:\msdchem\1\methods\Calibtration plot_Ace.M
Title : autoint1.e

Signal : TIC: 2_BR2W1.D\data.ms

eak #	R.T. min	first scan	max scan	last scan	PK TY	peak height	corr. area	corr. % max.	% of total
1	11.122	1172	1192	1215	BB 4	189220	7545588	2.05%	2.011%
2	12.656	1406	1452	1484	BB	26380862	367685282	100.00%	97.989%

Sum of corrected areas: 375230870

libtration plot_Ace.M Mon Jun 13 09:58:56 2016
