# Supplementary material for: Biofilm mediated synergistic degradation of hexadecane by a naturally formed community comprising Aspergillus flavus complex and Bacillus cereus group
Source: BMC Microbiol. 2019 Apr 29;19:84. doi: 10.1186/s12866-019-1460-4 (PMC6489202; doi:10.1186/s12866-019-1460-4)
Supplement: Supplementary file 2 — Chromatograms_GCMS 2. Balance chromatograms for residual HXD analyzed by GC-MS after 14 day incubation of cultures of the three communities (C1,C2 & C3) and counterparts of community C1. (PDF 132 kb) [file 12866_2019_1460_MOESM2_ESM.pdf]

60
MMI BaC R3

File :C:\msdchem\1\data\Madushika\3_BR3W1.D
Operator :
Acquired : 10 Jun 2016 12:50 using AcqMethod MADUSHIKA.M
Instrument : UOSJP GCMSD
Sample Name: 3_BR3W1
Misc Info : ERR
Vial Number: 3

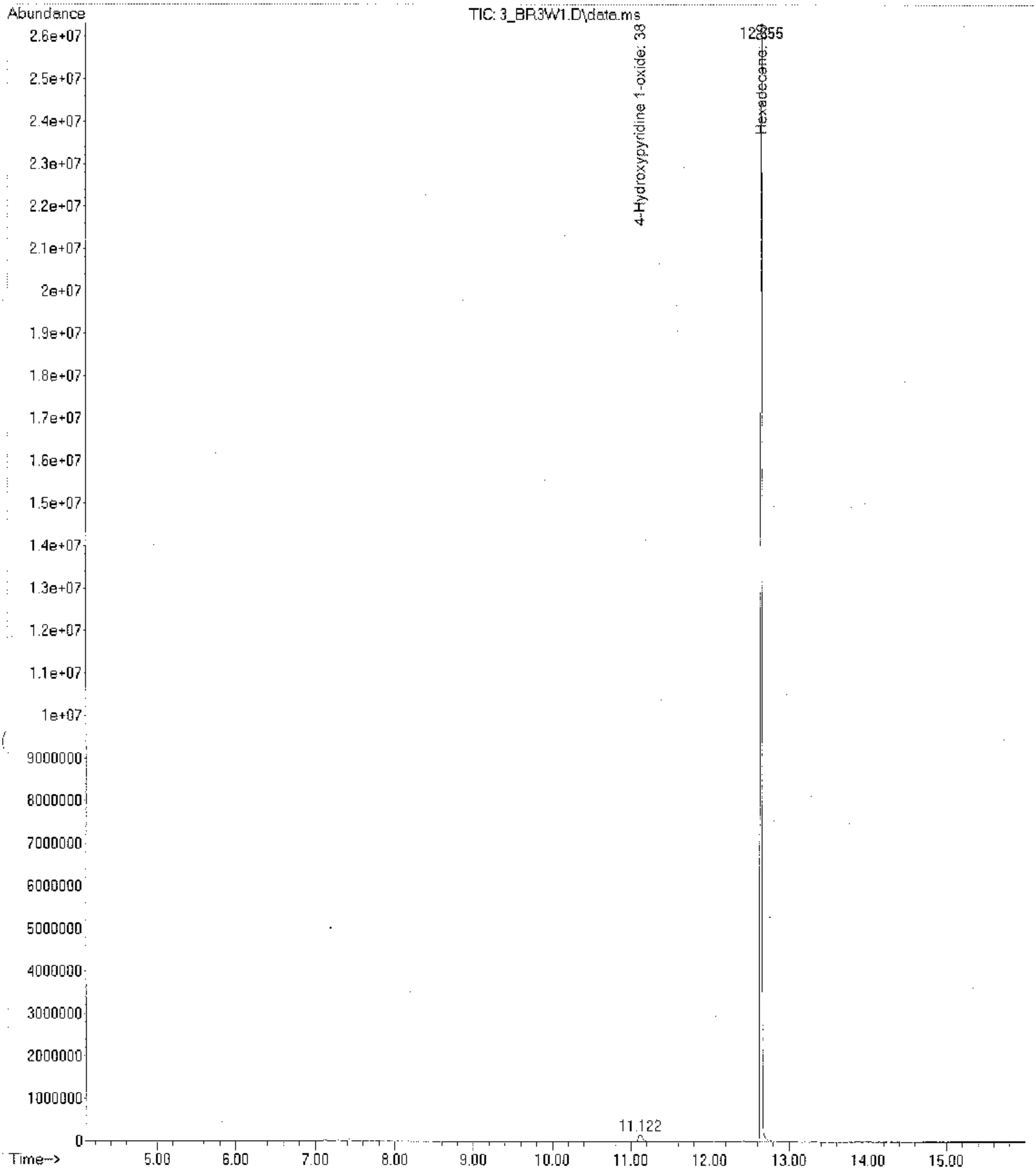

Area Percent Report

```
ata Path : C:\msdchem\1\data\Madushika\
ata File : 3_BR3W1.D
cq On    : 10 Jun 2016  12:50
perator  :
ample    : 3_BR3W1
isc      :
LS Vial  : 3   Sample Multiplier: 1

ntegration Parameters: autoint1.e
ntegrator: ChemStation

ethod    : C:\msdchem\1\methods\Calibtration plot_Ace.M
itle     : autoint1.e

ignal     : TIC: 3_BR3W1.D\data.ms
```

ak #	R.T. min	first scan	max scan	last scan	PK TY	peak height	corr. area	corr. % max.	% of total
	11.122	1165	1192	1217	BB 2	140944	5995186	1.65%	1.622%
	12.655	1406	1451	1483	BB	25829469	363602615	100.00%	98.378%

Sum of corrected areas: 369597801

ibtration plot_Ace.M Mon Jun 13 09:59:50 2016

61
MMI Bac' control

File :C:\msdchem\1\data\Madushika\4_BC1W1.D
Operator :
Acquired : 10 Jun 2016 13:11 using AcqMethod MADUSHIKA.M
Instrument : UOSJP GCMSD
Sample Name: 4_BC1W1
Misc Info : ERR
Vial Number: 4

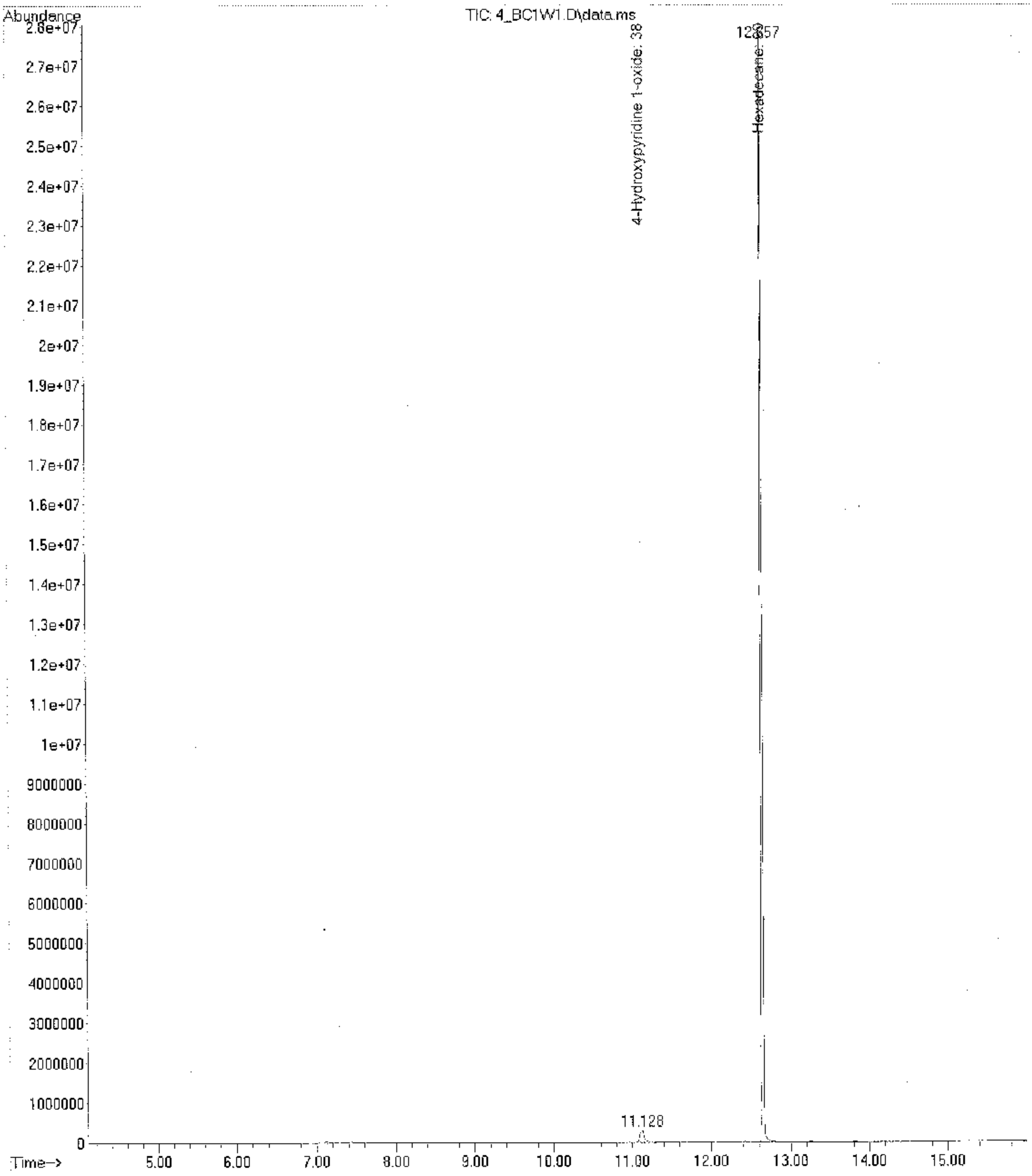

Area Percent Report

Data Path : C:\msdchem\1\data\Madushika\
Data File : 4_BC1W1.D
Acq On : 10 Jun 2016 13:11
Operator :
Sample : 4_BC1W1
Misc :
ALS Vial : 4 Sample Multiplier: 1

Integration Parameters: autoint1.e
Integrator: ChemStation

Method : C:\msdchem\1\methods\Calibtration plot_Ace.M
Title : autoint1.e

Signal : TIC: 4_BC1W1.D\data.ms

eak #	R.T. min	first scan	max scan	last scan	PK TY	peak height	corr. area	corr. % max.	% of total
1	11.128	1165	1193	1220	BB	276851	10738763	2.70%	2.626%
2	12.657	1406	1452	1484	BB	26653306	398233417	100.00%	97.374%

Sum of corrected areas: 408972180

libtration plot_Ace.M Mon Jun 13 10:01:06 2016

MMI Bac Control.

File :C:\msdchem\1\data\Madushika\5_BC2W1.D
Operator :
Acquired : 10 Jun 2016 13:32 using AcqMethod MADUSHIKA.M
Instrument : UOSJP GCMSD
Sample Name: 5_BC2W1
Misc Info : ERR
Vial Number: 5

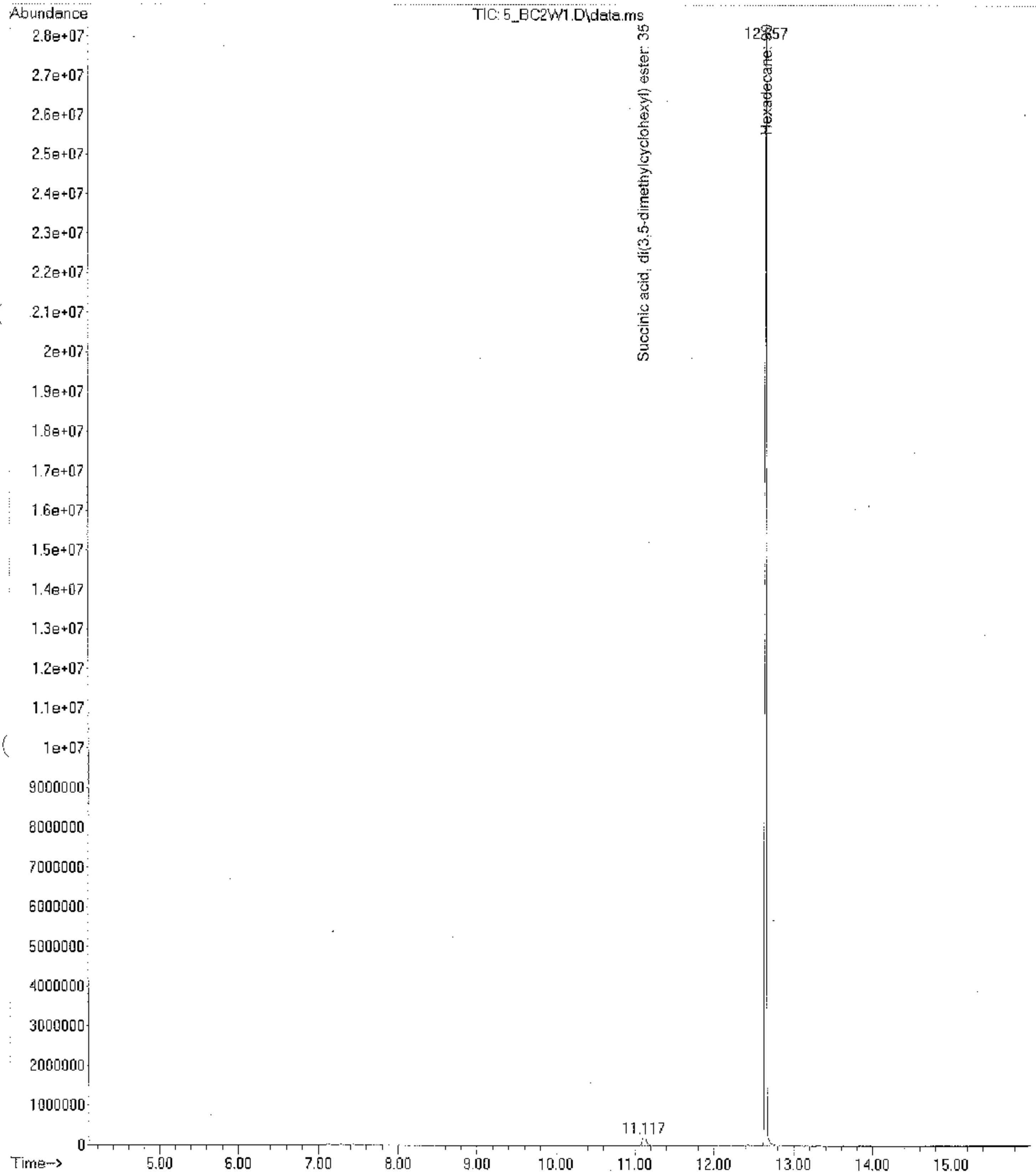

Area Percent Report

Data Path : C:\msdchem\1\data\Madushika\
Data File : 5_BC2W1.D
Acq On : 10 Jun 2016 13:32
Operator :
Sample : 5_BC2W1
Misc :
ALS Vial : 5 Sample Multiplier: 1

Integration Parameters: autoint1.e
Integrator: ChemStation

Method : C:\msdchem\1\methods\Calibtration plot_Ace.M
Title : autoint1.e

Signal : TIC: 5_BC2W1.D\data.ms

eak #	R.T. min	first scan	max scan	last scan	PK TY	peak height	corr. area	corr. % max.	% of total
1	11.117	1165	1191	1217	BB	192504	8276993	2.01%	1.970%
2	12.657	1405	1452	1484	BB	26848778	411770923	100.00%	98.030%

Sum of corrected areas: 420047916

libtration plot_Ace.M Mon Jun 13 10:02:24 2016

MM1 Bac Control after 2weeks

B C3W1

File :C:\msdchem\1\data\Madushika\6_BC3W1.D
Operator :
Acquired : 10 Jun 2016 13:53 using AcqMethod MADUSHIKA.M
Instrument : UOSJP GCMSD
Sample Name: 6_BC3W1
Misc Info :
Vial Number: 6

ERR

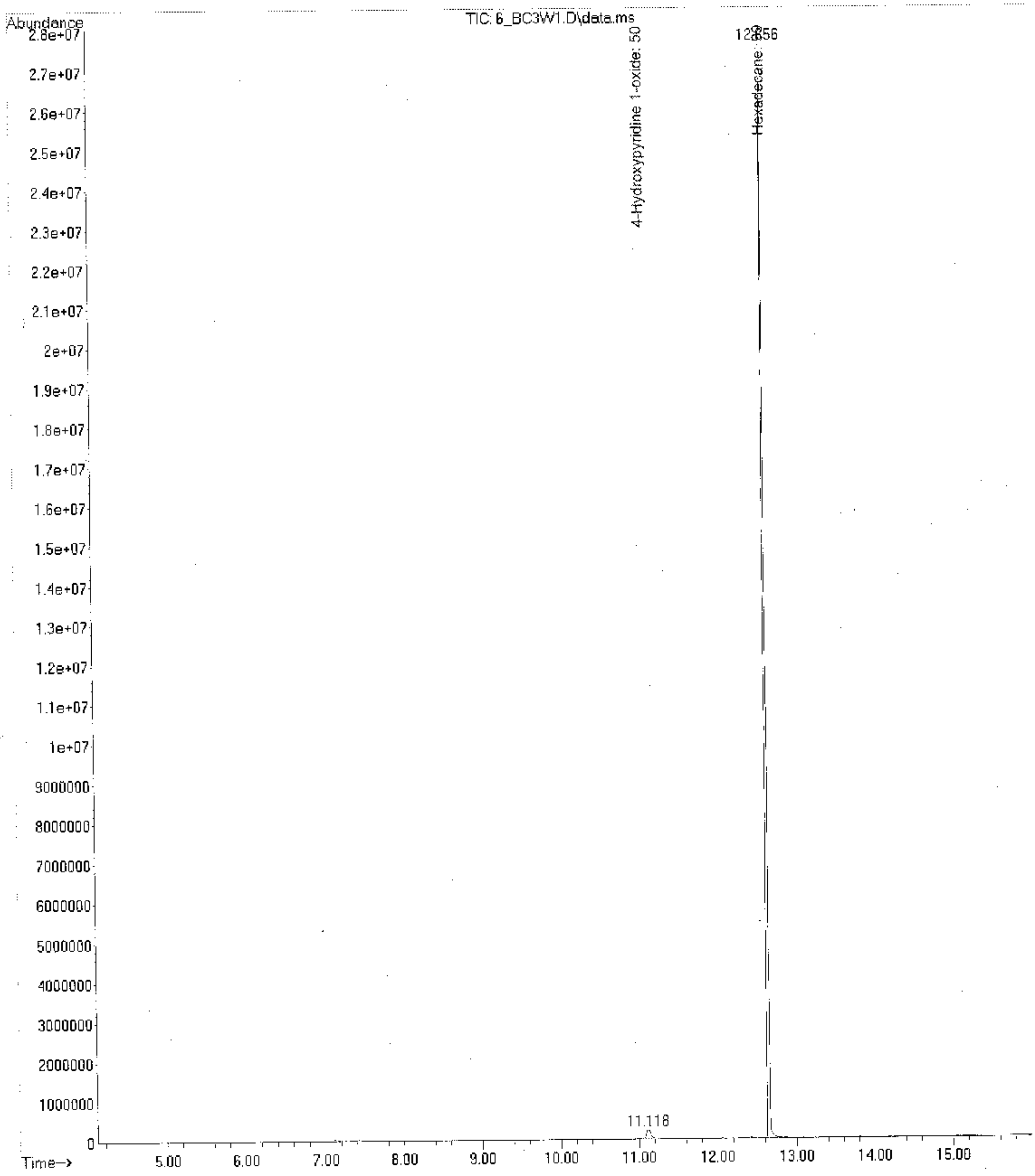

Area Percent Report

Data Path : C:\msdchem\1\data\Madushika\
Data File : 6_BC3W1.D
Acq On : 10 Jun 2016 13:53
Operator :
Sample : 6_BC3W1
Misc :
ALS Vial : 6 Sample Multiplier: 1

Integration Parameters: autoint1.e
Integrator: ChemStation

Method : C:\msdchem\1\methods\Calibtration plot_Ace.M
Title : autoint1.e

Signal : TIC: 6_BC3W1.D\data.ms

peak #	R.T. min	first scan	max scan	last scan	PK TY	peak height	corr. area	corr. % max.	% of total
1	11.118	1164	1191	1216	BB 2	212590	8656772	2.33%	2.280%
2	12.656	1406	1452	1482	BB	26477338	370955490	100.00%	97.720%

Sum of corrected areas: 379612262

Calibtration plot_Ace.M Mon Jun 13 10:03:25 2016
