# Supplementary material for: Biofilm mediated synergistic degradation of hexadecane by a naturally formed community comprising Aspergillus flavus complex and Bacillus cereus group
Source: BMC Microbiol. 2019 Apr 29;19:84. doi: 10.1186/s12866-019-1460-4 (PMC6489202; doi:10.1186/s12866-019-1460-4)
Supplement: Supplementary file 3 — Key to chromatograms in Additional files 1 and 2. (DOCX 12 kb) [file 12866_2019_1460_MOESM3_ESM.docx]

**Key to Chromatograms in additional files 1 & 2**

**Chromatograms in file 1**

| Chromatogram Numbers | Names | Description |
| --- | --- | --- |
| 1-3 | MM1R1, MM1R2, MM1R3 | Replicates of C1 community |
| 4-5 | MM1C2, MM1C3 | Replicates of negative controls of C1 community |
| 6 -8 | MM9R1, MM9R2, MM9R3 | Replicates of C2 community |
| 9-11 | MM9C1, MM9C2, MM9C3 | Replicates of negative controls of C2 community |
| 12-13 | MM10R1, MM10R3 | Replicates of C3 community |
| 14-16 | MM10C1, MM10C2, MM10C3 | Replicates of negative controls of C3 Community |
| 17-19 | CR1, CR2, CR3 | Replicates of fungus of C1 community |
| 20-22 | CC1,CC2,CC3 | Replicates of negative controls of fungus of C1 community |
| 23-24 | BR1, BR2 | Replicates of bacteria of C1 community |

**Chromatograms in file 2**

| Chromatogram Numbers | Names | Description |
| --- | --- | --- |
| 1 | BR3 | Replicate of bacterium of C1 community |
| 2-4 | BC1,BC2,BC3 | Replicates of negative controls of bacterium of C1 community |
